# Drug repositioning for immunotherapy in breast cancer using single-cell analysis

**DOI:** 10.1038/s41540-024-00359-z

**Published:** 2024-04-08

**Authors:** Elyas Mohammadi, Samira Dashti, Neda Shafizade, Han Jin, Cheng Zhang, Simon Lam, Mojtaba Tahmoorespur, Adil Mardinoglu, Mohammad Hadi Sekhavati

**Affiliations:** 1 Division of Neurogeriatrics, Department of Neurobiology, Care Sciences and Society, Karolinska Institutet, Stockholm, Sweden; 2 Department of Animal Science, Ferdowsi University of Mashhad, Mashhad, Iran; 3 Department of Internal Medicine, Mashhad University of Medical Science, Mashhad, Iran; 4 Science for Life Laboratory, KTH–Royal Institute of Technology, Stockholm, Sweden; 5 Cancer Research UK Cambridge Institute, University of Cambridge, Cambridge, UK; 6 Centre for Host-Microbiome Interactions, Faculty of Dentistry, Oral & Craniofacial Sciences, King’s College London, London, UK

**Keywords:** Cancer, Immunology

## Abstract

Immunomodulatory peptides, while exhibiting potential antimicrobial, antifungal, and/or antiviral properties, can play a role in stimulating or suppressing the immune system, especially in pathological conditions like breast cancer (BC). Thus, deregulation of these peptides may serve as an immunotherapeutic strategy to enhance the immune response. In this meta-analysis, we utilized single-cell RNA sequencing data and known therapeutic peptides to investigate the deregulation of these peptides in malignant versus normal human breast epithelial cells. We corroborated our findings at the chromatin level using ATAC-seq. Additionally, we assessed the protein levels in various BC cell lines. Moreover, our in-house drug repositioning approach was employed to identify potential drugs that could positively impact the relapse-free survival of BC patients. Considering significantly deregulated therapeutic peptides and their role in BC pathology, our approach aims to downregulate B2M and SLPI, while upregulating PIGR, DEFB1, LTF, CLU, S100A7, and SCGB2A1 in BC epithelial cells through our drug repositioning pipeline. Leveraging the LINCS L1000 database, we propose BRD-A06641369 for B2M downregulation and ST-4070043 and BRD-K97926541 for SLPI downregulation without negatively affecting the MHC complex as a significantly correlated pathway with these two genes. Furthermore, we have compiled a comprehensive list of drugs for the upregulation of other selected immunomodulatory peptides. Employing an immunotherapeutic approach by integrating our drug repositioning pipeline with single-cell analysis, we proposed potential drugs and drug targets to fortify the immune system against BC.

## Introduction

Breast cancer (BC), in general, has low immunogenicity^1^; however, immune activation can be pivotal in BC prognosis and treatment^2^. Cancer immunotherapy, also known as immuno-oncology, is a form of cancer treatment that educates, boosts or inhibits the immune system components to prevent, control, or eliminate cancer^3^. Antimicrobial and immunomodulatory peptides (AMPs) are a part of the innate immune system^4^. AMPs may directly interact with pathogens and/or malignant cells or indirectly modulate the immune system leading to the elimination of target cells^4,5^. On the other hand, AMPs that are capable of eliminating pathogens may promote cancer progression^6^. Therefore, understanding the cancer-specific response of AMPs and exploring methods to enhance or suppress it can pave the way for immunotherapy in precision medicine.

To better understand the immunotherapy targets, various techniques are deployed to assess gene expression^7^ including conventional bulk transcriptomics and single-cell transcriptomics (scRNA-seq)^8^. Bulk transcriptomics measures the average of gene expression across all cells. scRNA-seq deals with gene counts in each cell; hence, it provides the opportunity to categorize and annotate the cell types based on their expression profiles. Breast cancer is heterogeneous and also comprises—beyond malignant cells—stromal and immune tumor-associated cells. Although bulk transcriptomics still has its own advantages in cancer immunotherapy^8^, for precision onco-immunotherapy scRNA-seq provides higher resolution for both malignant and immune cell profiling^8,9^.

Single-cell studies have improved drug target and biomarker discovery in immunotherapy^10–12^, while finding the potential drugs for these targets is still challenging^13^. Drug repositioning against immunotherapy targets explores the therapeutic use of existing clinically approved, off-patent drugs. These chemicals have known modes of action and targets for another indication. Thus, exploring them minimizes the cost of therapy, time and risk^14–17^.

Deregulation of immunomodulatory peptides is considered as an independent immunotherapy treatment or auxiliary to obtain better results through combination therapy^18,19^. In this study, we aimed to investigate the endogenous expression of, to the best of our knowledge, all so far known human antimicrobial and immunomodulatory peptides in BC^6^. To do so, we utilized BC and normal breast scRNA-seq data in addition to Assay for Transposase-Accessible Chromatin using sequencing (ATAC-seq) data from BC cell lines. This in turn improved the resolution of our analysis as we could extract and compare malignant versus nonmalignant epithelial cells at different omics levels (i.e., chromatin and gene levels). Based on the role of upregulated immunomodulatory peptides in cancer biology, we proposed drugs that can deregulate a subset of these peptides.

## Results

### **Extracting the transcriptomics profiles of epithelial cells**

**Fig. 1:**
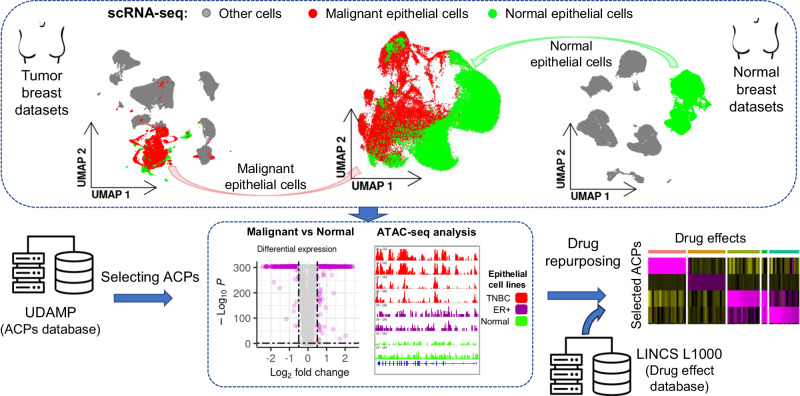
scRNA-seq provides single-cell resolution to compare different cell states. Using scRNA-seq, it is possible to extract the desired cell types in condition A (top left panel: malignant epithelial cells) and compare them to the same or different cell types in condition B (top right panel: nonmalignant epithelial cells). We studied the deregulation of endogenous therapeutic peptides derived from the University of Debrecen Antimicrobial and Immunomodulatory Peptide (UDAMP) database at different omics levels in BC epithelial cells. Next, we investigated the available perturbagens on BC cell lines in the Library of Integrated Network-based Cellular Signatures (LINCS) database and proposed candidate drugs that can change the expression of these peptides to eliminate the malignancy.

In this study, we aimed to investigate the deregulation of antimicrobial and immunomodulatory peptides in malignant versus nonmalignant human breast epithelial cells at different omics levels. Hence, we would be able to predict the potential candidate drugs that can change the expression of these peptides in favor of patients (Fig. 1). We retrieved two publicly available scRNA-seq datasets (details in Data Availability) from 13 normal^20^ and 20 BC patients^1^ (3 HER2 + , 9 ER+ and 8 TNBC subtypes). The cells in the BC dataset were previously annotated for cell types. Besides, the epithelial cells, as the most common site for the development of BC^21^, were categorized into malignant and nonmalignant cells. The classification of epithelial cells was done by identifying evidence for large-scale chromosomal copy number variations^22^. This in turn increased the resolution of our analysis, resulting in 24489 malignant epithelial cells extracted from tumor samples.

To acquire the normal epithelial cells, first we classified all cells using an unsupervised clustering method (see material and methods). Next, using Human Primary Cell Atlas (HPCA)^23^ and Blueprint Epigenomics (BP)^24^ as two previously annotated cell references, we could investigate the distribution of label scores among cells, (Supplementary Fig. 1A). We observed distinct scoring profiles among cells, demonstrating unambiguous concordance between cells and reference annotations and allowing us to accordingly assign labels to cells. In order to investigate the probable outliers in each labeled cluster, we examined the delta values for each cell, i.e., the difference between the score for the assigned label and the median across all labels for each cell. Few outliers were observed and the label assignments were deemed to be accurate (Supplementary Fig. 1B). Afterwards, to compare the concordance between the two independent methods of unsupervised clustering and cell label assignment, we investigated the distribution of cell labels in each cluster. We observed strong agreement between the two methods (Supplementary Fig. 1C). Accordingly, we assigned the determined annotations to clusters (Supplementary Fig. 1D) and subsequently extracted the 21698 normal epithelial cells for downstream analysis.

### Differential gene expression analysis to track AMPs

**Fig. 2:**
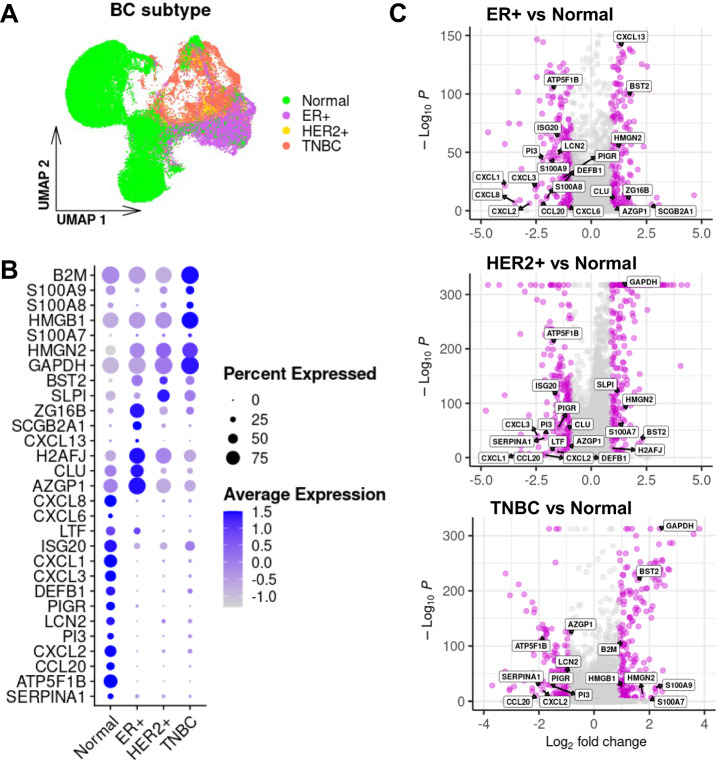
Immunomodulatory peptdeis are deregulated across BC and normal epithelial cells. **A** The UMAP from integrated data. **B** Expression of upregulated AMPs across different BC subtypes. **C** Up and downregulated AMPs in different BC subtypes versus normal epithelial cells.

For joint analysis of data from multiple scRNA-seq datasets (e.g., differential gene expression analysis between malignant and normal epithelial cells), we need to harmonize them into a single reference in order to remove technical artifacts. To do so, we used the canonical correlation analysis (CCA) for scRNA-seq data integration^25^. We identified cell pairwise correspondences between single cells in two sets, henceforth termed “anchors”. Anchors represent the cellular relationships across datasets, which can be used for scRNA-seq data integration by recovering the matching cell states (See material and methods)^25^. The integrated data is illustrated in Fig. 2A. A clear seperation of malignant and normal epithelial cells can be seen.

After data integration, we performed differential gene expression (DEG) analysis (BC subtypes vs normal) to explore deregulated genes encoding immunomodulatory peptides in BC (Fig. 2B, C). AMPs were retrieved from UDAMP^6^ (Supplementary Table 1). Among the AMPs in UDAMP datasets, we identified 29 deregulated immunomodulatory genes, 14 downregulated, and 15 upregulated in BC subtypes (Fig. 2B). We showed the significancy (-Log_10_P) and the log of fold changes of deregulation of these genes in Fig. 2C.

Eight immunomodulatory peptides were selected for downstream analysis. The expression of PIGR, DEFB1, LTF, CLU, SCGB2A1 and S100A7, while having a positive role in cancer elimination, were either downregulated or not changed in all or some of the BC subtypes (Table 1). The role of SLPI and B2M in cancer progression and metastasis has been shown before and theyr are known as promissing drug targers^26^. These two peptides were upregulated in some of the BC subtypes (Table 1).

**Table 1:** Deregulation of immunomodulatory peptides can be an immunotherapy strategy

Candidate peptides	Therapeutic role	Deregulation			Association in BC biology	DR/ Direction	Ref
		ER +	HER2 +	TNBC			
SERPINA1	Antimicrobial	NA	—	—	Prognostic marker (favorable)	No	^61^
ATP5F1B	Antibacterial,	—	—	—	Prognostic marker (unfavorable)	No	^62^
CCL20	Antibacterial	—	—	—	Cancer progression and migration	No	^63^
CXCL2	Antibacterial	—	—	—	Metastasis	No	^64^
PI3	Antibacterial	—	—	—	Recurrence	No	^65^
LCN2	Antibacterial	—	NA	—	Tumorigenesis and progression	No	^66^
PIGR	Antimicrobial	—	NA	—	Survival	Yes/Up	^67^
DEFB1	Antibacterial	—	—	NA	Tumor-suppressor	Yes/Up	^68^
CXCL3	Antibacterial	—	—	NA	Tumor cell migration and invasion	No	^69^
CXCL1	Antibacterial	—	—	NA	Advanced cancer stage, lymph node metastasis and poor survival	No	^70^
ISG20	Antiviral	—	—	NA	Tumor progression and metastasis	No	^71^
LTF	Antimicrobial	NA	—	NA	Survival	Yes/Up	^72^
CXCL6	Antibacterial, Antiparasitic	—	NA	NA	Metastasis	No	^73^
CXCL8	Antibacterial	—	NA	NA	Metastasis	No	^73^
AZGP1	Antimicrobial	+	—	—	Prognostic marker (favorable)	No	^74^
CLU	Antimicrobial	+	—	NA	Regulates the infiltration of immune cells	Yes/Up	^75^
H2AFJ	Antibacterial, Antifungal	NA	+	NA	Relapse-free survival	No	^76^
CXCL13	Antibacterial	+	NA	NA	Tumor progression	No	^77^
SCGB2A1	Antimicrobial	+	NA	NA	Less aggressive behavior and a more favorable outcome	Yes/Up	^78^
ZG16B	Antimicrobial	+	NA	NA	Tumor marker	No	^79^
SLPI	Antibacterial, Antifungal, Antiviral	NA	+	NA	Gene target to stop the metastasis	Yes/ Down	^26^
BST2	Antiviral	+	+	+	Invasiveness, poor Survival, disease severity	No	^41^
GAPDH	Antibacterial, Antifungal	NA	+	+	Cancer cell proliferation	No	^42^
HMGN2	Antibacterial, Antiviral, Antifungal	+	+	+	Metastasis	No	^80^
S100A7	Antibacterial	NA	+	+	Decrease migration and proliferation; wound healing in breast cancer	Yes/Up	^48^
HMGB1	Antimicrobial	NA	NA	+	Cancer progression and drug resistance	No	^81^
S100A8	Antifungal	—	NA	NA	Progression, poor prognosis and overall survival	No	^82^
S100A9	Antibacterial	—	NA	+	Progression, poor prognosis and overall survival	No	^82^
B2M	Antimicrobial	NA	NA	+	Tumor cell growth/Gene target	Yes/ Down	^83^

Based on the association of nine upregulated immunomodulatory peptides in cancer biology, we determined a strategy for drug repositioning.

The selected AMPs are a part of the innate immune system with a therapeutic effect against pathogens (Table 1). However, their role as immunomodulatory peptides against cancer is variable. We categorized these nine AMPs into two groups based on their association with cancer biology. The first group contains the genes that are connected with invasiveness, poor survival, disease severity, cancer cell proliferation, and metastasis. We aimed to decrease the expression of genes in this group using drug repositioning. The second group contains the genes associated with less aggressive behavior, more favorable outcomes in cancer and relapse-free survival. We attempted to elevate their expression using drug repositioning. The rest of the genes in Table 1 are either cancer markers or show dual behavior in different malignancies. We did not consider these genes for drug repositioning.

### Chromatin accessibility analysis

While chromatin accessibility is not the sole determinant of gene deregulation, it contributes valuable insights into the potential sources of variation in gene expression under both normal and pathological conditions. To complement our scRNA-seq data findings at the chromatin level, we acquired ATAC-seq data for four distinct breast cancer (BC) epithelial cell lines: two triple-negative breast cancer (TNBC) cell lines (MDA-MB-231 and MDA-MB-436) with repetitions^27^, one estrogen receptor-positive BC cell line (MCF7) with repetitions^28^ and two normal MCF-10A breast epithelial cell lines^29,30^.

**Fig. 3:**
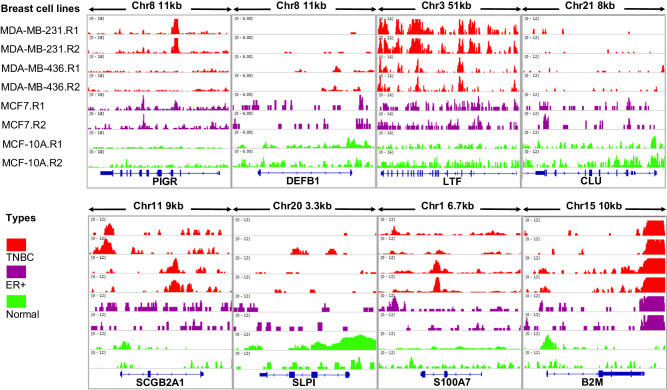
Chromatin accesibility may explain the deregulation of genes. An illustration of crhomatin accisibility using ATAC-seq data for four distinct breast cancer (BC) cell lines including two triple-negative breast cancer (TNBC) cell lines (MDA-MB-231 and MDA-MB-436) with repetitions^27^, one estrogen receptor-positive BC cell line (MCF7) with repetitions^28^ and two normal MCF-10A breast epithelial cell lines^29,30^.

Among the immunomodulatory peptides chosen for drug repositioning, the accessibility peaks for DEFB1 and CLU were notably higher in normal cells (Fig. 3), elucidating their downregulation in malignant epithelial cells compared to normal cells (Table 1, Fig. 2). LTF exhibited similar peak patterns for the ER + BC cell line compared to normal (Fig. 3), supporting the observation of nonsignificant deregulation of LTF in the ER+ subtype. Elevated peaks for SCGB2A1 in the ER+ cell line compared to normal further substantiate the upregulation of this gene at the transcriptomics level. Regarding S100A7, the higher peaks in ATAC-seq data for TNBC and a similar pattern in ER+ versus normal (Fig. 3) support the upregulation and nonsignificant gene changes for TNBC and ER + BC subtypes, respectively (Table 1).

### Proteomics analysis

In order to investigate the distribution of protein expression of selected AMPs across different BC subtypes, we used the proteomics profiles of 26 BC cell lines. We performed the dimentionality reduction and illustrated the seperation of four HER2 + , seven ER + , and fifteen TNBC using UMAP visualization (Supplementary Fig. 2A). Among the selected AMPs, only B2M, SLPI, CLU and LTF were available in proteomics data. We showed significant upregulation of B2M, SLPI and LTF while downregulation of CLU proteins in TNBC compared to HER2+ and ER + BC subtypes.

### Drug repositioning

**Fig. 4:**
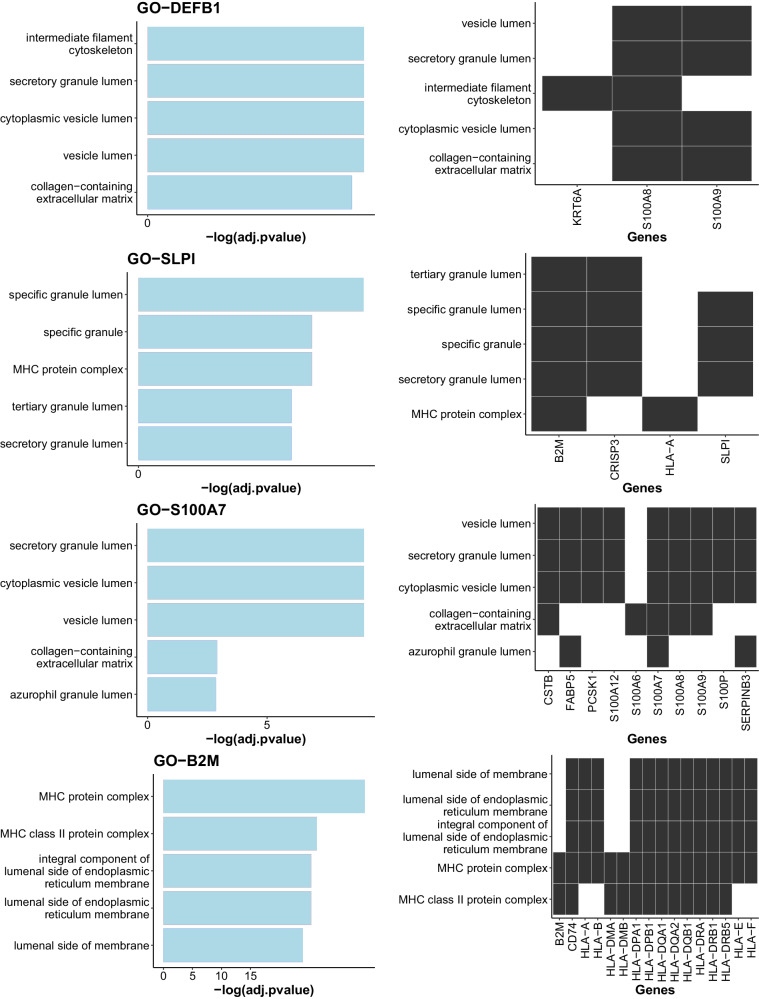
GO pathway analysis. Investigating possible immunological pathways that we may intervene by deregulating selected AMPs through drug repositioning.

Our goal of drug repositioning is to deregulate the candidate AMPs based on their role in cancer biology. Hence, we need to know which pathways we may intervene during this process. In order to study the functional aspects of candidate APMs, we performed a correlation followed by Gene Ontology (GO) analysis. The correlation was calculated among the top 3000 variable genes in the scRNA-seq dataset. The top 50 significantly correlated genes with each candidate AMP were extracted if the correlation was more than 0.3 and adjusted pvalue (Bonferroni correction) awas less than 0.05 (Supplementary Table 2). Next, these gene sets were applied to GO pathway analysis by selecting the top five significantly enriched terms (p-value < 0.05, Benjamini-Hochberg correction). Among the AMPs with identified GO terms in Fig. 4, we found that B2M is correlated with HLA genes and consequently participates in the MHC protein complex. On the other hand, SLPI is correlated with B2M and enrolls in MHC pathway (Supplementary Fig. 2). Thus, we need to be cautious about downregulation of B2M and SLPI in a way that repurposed drugs only target these two genes and not the correlated MHC genes.

To explore the effect of previously approved drugs against candidate AMPs in BC, we extracted all available perturbagen data for all BC cell lines in the LINCS L1000 database. LINCS L1000 is an actively growing collection of gene expression profiles for thousands of perturbagens at a variety of time points, doses, and cell lines^31^. Among the BC cell lines in LINCS L1000, we chose MCF7 for downstream analysis. The rationale behind our selection was the number of available perturbagens for each BC cell line. According to the data availability in LINCS L1000, among the BC cell lines, the number of perturbagens for MCF7 is considerably higher than other cells (Supplementary Fig. 2C). Hence, it provides a great opportunity to discover potential drugs out of ~30000 perturbagens that can be repurposed in our study.

**Fig. 5:**
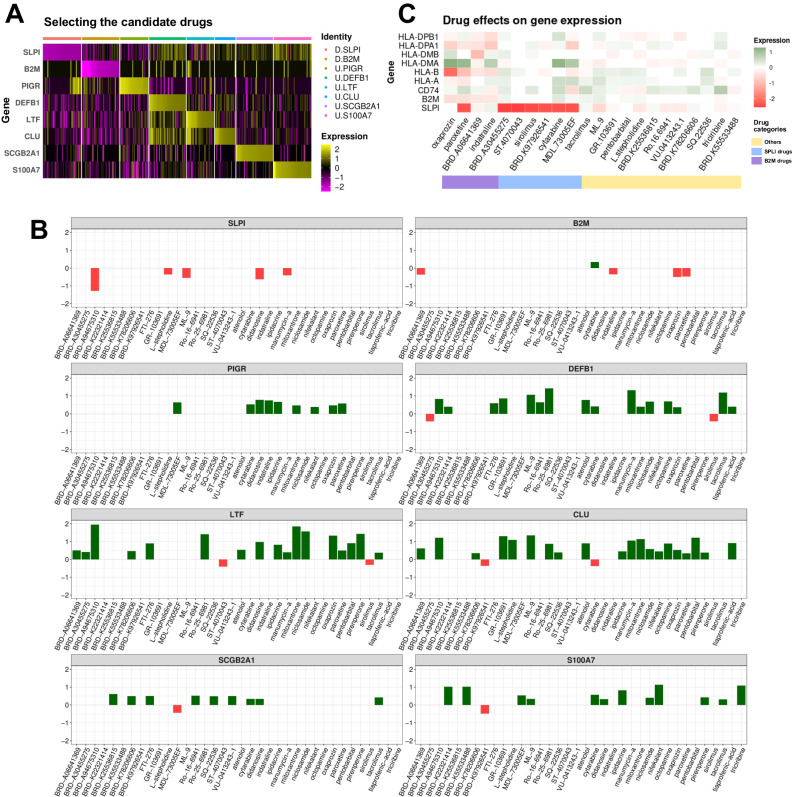
Drug repositioning can find new indications for previously approved drugs. **A** The heatmap depicts the impact of selected drugs on candidate genes. In the legends, “D” and “U” preceding the gene names represent the planes for drug repositioning, signifying downregulation and upregulation, respectively. **B** The drugs were filtered by excluding those with contradictory impacts on each other, aligning with the drug repositioning plane. **C** The figure illustrates the effect of drugs that downregulate B2M and/or SLPI on MHC complex-associated genes.

We computed the LFC values for all MCF7 drug perturbagens versus DMSO as control (see material and methods). Next we extracted the LFC values of the candidate AMPs across all ~30000 perturbagens. We identified the top 50 perturbagens capable of deregulating candidate AMPs in favor of BC patients (Fig. 5A) and compiled its metadata and LFC values in Supplementary Table 3 and 4, respectively. While presenting this list illuminates the alternative effects of established drugs on new targets, it is crucial to consider the impact of these drugs on other selected AMPs to mitigate potential negative off-target effects. Consequently, we refined our selection by filtering drugs that exhibit a beneficial effect on the deregulation of one AMP while compromising the effect on others in BC patients (Fig. 5B). This process allowed us to propose potential drugs for repurposing, specifically tailored to deregulate immunomodulatory peptides in favor of BC patients.

Based on the correlation of B2M and SLPI with genes associated with the MHC complex (Fig. 4), we generated plots illustrating the impact of drugs selected for the downregulation of B2M and/or SLPI on the available MHC complex-related genes. Additionally, we included drugs chosen for other AMPs that show a positive role in the downregulation of B2M and/or SLPI. Among the drugs targeting B2M, Oxaprozin, Paroxetine, and Indatraline, along with Cytarabine and MDL-73005EF targeting SLPI, notably decreased the expression of HLA-DMA. However, with the exception of BRD-A30455275 and Cytarabine, the remaining drugs did not consistently demonstrate downregulation for MHC complex genes. Consequently, for B2M, BRD-A06641369, and for SLPI, ST-4070043 and BRD.K97926541, illustrated the least conflict with MHC complex genes (Fig. 5C).

## Discussion

BC arises from breast epithelial cells that acquire specific genetic variations leading to the subsequent loss of tissue homeostasis^32^. However, not all of the epithelial cells are malignant in a cancerous tissue^1^. To raise the accuracy of our analysis, we extracted only the transcriptomics profiles of malignant epithelial cells in cancer patients and subsequently compared them to normal epithelial cells extracted from healthy patients. The same approach has been frequently used in different cancer studies using InferCNV method^33–35^. This gave us the opportunity to investigate the changes in the expression of, to the best of our knowledge, all so far known human endogenous AMPs^6^ in malignant compared to normal breast epithelial cells.

We showed the overexpression of eight immunomodulatory peptides in gene level of BC epithelial cells versus normal and supported these results in chromatin level. The elevated expression of these AMPs in different cancer is also reported previously^26,36–39^. However, the therapetuic use of these peptide against BC by manipulationg their expression is yet to be explored.

Pavlicevic et al.^19^ introduced the AMPs as a promising source for drug discovery. These components are a complex class of bioactive peptides with diverse effects on both innate and adaptive immunities. The main focus of Kumar et al.^6^ on data curation of AMPs in UDAMP database was on their antimicrobial, antifungal and antiviral features. Hence, we used this source to study the elevated immunomodulatory peptides in BC to see if they are potential to be anticancer, cancer driver or ineffective in cancer progression (Table 1).

AMPs may have single or multiple functions^6,40^ and conflicts among these roles can arise. For instance, an antimicrobial peptide may promote the cell growth of malignant cells (Table 1). For instance, certain antimicrobial peptides (AMPs) may unexpectedly stimulate the growth of malignant cells, as mentioned in Table 1. For instance, B2M, SLPI, BST2, GAPDH, S100A8, S100A9, and HMGB1, despite their beneficial roles in various infections (Supplementary Table 1), they contribute to cancer cell proliferation, invasiveness, reduced survival, and increased disease severity across different cancers^18,41–46^. However, not all of these peptides can be considered viable drug targets, owing to their involvement in cellular homeostasis. For instance, GAPDH plays a crucial role in glycolysis, and its downregulation can impede the glycolytic cycle, affecting cellular metabolism in both malignant and healthy cells^47^.

To effectively target AMPs, understanding the actions of immunomodulatory peptides in breast malignant epithelial cells and devising strategies to deregulate these peptides in favor of patients is essential. S100A7 exhibits dual behavior in various malignancies. In cervical cancer and BC^37,38,48^ S100A7 promotes increased and decreased cancer cell proliferation, respectively, while also functioning as an antibacterial peptide^6^. B2M emerges as a promising therapeutic target for cancer in various solid tumors, including human lung, breast, renal, and prostate cancers^39^. In addition, SLPI is a potential target for inhibiting metastasis of triple-negative breast cancers^49^. Additionally, SLPI represents a potential target for inhibiting metastasis in triple-negative breast cancers. The role of other AMPs in pathology of cancer is summerized in Table 1.

The application of the LINCS L1000 project^31^ in drug repositioning for immunotherapy has been previously explored^50,51^. While various studies have attempted to repurpose non-human immunomodulatory peptides^52^ and immunomodulatory drugs for different disorders^53^. our research represents the effort to focus on repurposing drugs to modulate the expression of human immunomodulatory peptides for the elimination of BC.

The anticancer effects of several drugs depicted in Fig. 5C have already been substantiated against breast cancer (BC). Sirolimus, for instance, has been identified as a potentially effective treatment option for patients with hormone receptor-positive advanced breast cancer^54^. Kim et al. (2010) demonstrated the inhibitory effect of oxaprozin on the proliferation of MCF7 cell lines^55^. Additionally, Cho et al. (2019) established that paroxetine, an antidepressant drug, induces apoptosis in MCF-7 cells^56^. Wang et al. (2022) provided evidence that sodium pentobarbital suppresses the growth of BC cells^57^.

There are several steps that can enhance the robustness of our study findings. These include conducting wet lab validations and utilizing a multiomics platform to capture both scRNAseq and scATACseq data from the same patients. It is essential to note that results obtained from cancer cell lines may not be entirely translatable to human studies.

In summary, the safety profiles of all proposed drugs and the anticancer features of some have already been verified. Further investigation into the remaining repurposed drugs holds the potential for effective treatment in breast cancer patients.

## Methods

### Data collection and filtering

The processed data for 20 BC patients was obtained from the study conducted by Wu et al.^1^. Cells were previously filtered based on percentage of mitochondrial genes, high and low number of features and doublets^1^. In addition, epithelial cells were distinguished as normal or malignant. The classification of epithelial cells in these patients was performed using the InferCNV R package^33^. InferCNV can identify evidence for large-scale chromosomal copy number variations by exploring expression intensity of genes across positions of the genome in comparison to the average or a set of reference normal cells^33^. The processed data for 13 normal breast samples was retrieved from the study conducted by Pal et al.^20^. The percentage of mitochondrial genes in each cell was calculated using PercentageFeatureSet from Seurat package (Version 4.1.0)^58^. According to Pal et al. 2021, no more than 20% mitochondrial reads were generally allowed per cell, although the upper limit was increased as high as 40% for a small number of libraries. Cells with exceptionally high numbers of reads or genes detected were also filtered to minimize the occurrence of doublets. An average of 5000 cells per sample remained after this quality filtering.

Normalized ATAC-seq data for four BC cell lines were retrieved. These cells included two TNBC cell lines, i.e MDA_MB_231 and MDA_MB_436 with one repetition^27^, MCF7 as an ER + BC cell line with a repetition^28^, and two MCF10A as normal breast epithelial cell lines^29,30^. The Integrative Genomics Viewer (IGV) was employed to visualize the chromatin accessibility of candidate genes across six breast cancer (BC) cell lines and two control samples. Proteomics profiles of breast cancer cell lines (4 HER2 + , 7 ER + , and 15 TNBC; refer to Data Availability) were sourced from the Cancer Cell Line Encyclopedia (CCLE)^59^.

Transcriptomics data was processed in the Seurat R package (Version 4.1.0). The top 3000 features by variance across the datasets were selected using variance-stabilizing transform (VST) method and scaled using the default parameters of the ScaleData function of Seurat. Finally, dimensionality reduction was performed based on these scaled features using default parameters of RunPCA and RunUMAP functions of the Seurat^58^. The principal components, accounting for the predominant portion of variation in the data, were chosen through the utilization of the ElbowPlot function within the Seurat R package. We used a Shared Nearest-neighbor (SNN) graph construction method using FindNeighbors function in addition to a modularity optimization of SNN results using FindClusters function of the Seurat R package with default parameters to do an unsupervised clustering and categorize the normal breast single cell dataset. We plotted the reduced dimentions using Uniform Manifold Approximation and Projection (UMAP).

### Cell annotation

The BC dataset was previously annotated. To annotate the single cells from normal breast samples in the query set, we employed the SingleR package (Version 1.8.1) with the Human Primary Cell Atlas (HPCA)^23^ and Blueprint Epigenomics (BP)^24^ as two pre-annotated references. The integration and processing of these references were performed using Seurat. Subsequently, the HPCA and BP references were merged, and their labels were transferred to the query dataset. To illustrate the annotation results, PlotScoreHeatmap of the SingleR R package shows the assigned label scores to each cell. The package pheatmap (version 1.0.12) was used to depict the consistency between unsupervised clustering and supervised cell annotation. PlotDeltaDistribution plots the distribution of delta values for each cell, i.e., the difference between the score for the assigned label and the median across all labels for each cell. Finally, the results from all these steps were transferred to the defined clusters of normal breast samples.

### Data integration

Normal and malignant epithelial cells were extracted from normal and BC datasets and then merged and normalized using the LogNormalization method. Next, top 5000 variable features between two datasets were defined by the FindIntegrationFeatures function for data integration. Based on these variable genes we used the FindIntegrationAnchors function to determine the cell pairwise correspondences across single cells datasets^25^. Finally, the IntegrateData was used to perform data integration by pre-computed anchor set.

### Differential gene expression

Differential gene expression analysis between malignant and normal epithelial cells was done on integrated object. DEGs were defined using FindMarkers function by pairwise comparison of BC subtypes and normal epithelial cells, separately. In order to remove the batch effects in DEG computation, the MAST method^60^ was considered by setting the *latent.vars* parameter to patients in BC and normal datasets. Next, all so far known immunomodulatory peptides were extracted from University of Debrecen Antimicrobial and Immunomodulatory Peptide (UDAMP) database^6^. Deregulation of these genes were investigated among DEGs and illustrated by EnhancedVolcano plot (Version 1.12.0). Afterwards, the role of deregulated AMPs in BC biology were determined; i.e the genes that are in the favor of cancer progression and the genes associated with positive outcome and cancer elimination.

### Drug repositioning

The metadata for all BC cell lines in LINCS L1000 database were extracted and categorized based on BC subtypes i.e, TNBC, HER2+ and ER + . Number of available perturbagens with 10 μm dose and 24 hours treatment duration for each BC cell line was investigated by the Slinky R package (Version 1.12.0). The BC cell line with the most number of perturbagens based on aforementioned criteria was selected for downstream analysis. The control expression profiles were considered as treated with DMSO with the same dose and duration criteria. Next, the LFCs for the effect of each drug on the expression profile of the selected BC cell line was calculated. To do so, first the mean of gene expression for the effect of each drug across the repetitions was calculated for both control and treatment. Next, we computed the log2 of the mean of gene expressions and finally, the log transformed results for control and treatments were subtracted.

We selected a subset of AMPs which have a positive or negative role in BC elimination. The Log Fold Changes (LFCs) of the candidate AMPs across all drugs were extracted from the log fold change matrix. The profiles were categorized to the drug repositioning purposes based on the role of these genes in cancer biology i.e, “Down” (downregulation drug repositioning strategy) and “Up” (upregulation drug repositioning strategy). For the genes that should be up or downregulated through drug repositioning, the profiles were sorted descending and ascending, respectively. Hence, top 20 drugs that upregulate or downregulate the genes were chosen based on the purpose of drug repositioning. Finally, the effect of the proposed drugs on deregulation of genes were plotted.

### Reporting summary

Further information on research design is available in the Nature Research Reporting Summary linked to this article.

### Supplementary information

Reporting summary

Supplementary Files

## Data Availability

The scRNA-seq data for breast cancer and normal patients are publicly available through the Gene Expression Omnibus under accession numbers GSE176078 and GSE161529, respectively. The ATAC-seq data for BC cell lines used in this study are publicly available through the Gene Expression Omnibus under accession numbers GSE114964, GSE174152, GSE89013, and GSE121370.

## Code availability

We uploaded the scripts in R programming language to GitHub: https://github.com/ElyasMo/Drug.rep_BC.
