# Supplementary material for: Drug repositioning for immunotherapy in breast cancer using single-cell analysis
Source: NPJ Syst Biol Appl. 2024 Apr 8;10:37. doi: 10.1038/s41540-024-00359-z (PMC11001976; doi:10.1038/s41540-024-00359-z)
Supplement: Supplementary file 1 — Reporting summary [file 41540_2024_359_MOESM1_ESM.pdf]

nature portfolio

Corresponding author(s): Mohammad Hadi Sekhavati
Elyas Mohammadi

Last updated by author(s): Mar 5, 2024

# Reporting Summary

Nature Portfolio wishes to improve the reproducibility of the work that we publish. This form provides structure for consistency and transparency in reporting. For further information on Nature Portfolio policies, see our Editorial Policies and the Editorial Policy Checklist.

## Statistics

For all statistical analyses, confirm that the following items are present in the figure legend, table legend, main text, or Methods section.

| n/a | Confirmed | |
|---|---|---|
| ☐ | ☒ | The exact sample size (*n*) for each experimental group/condition, given as a discrete number and unit of measurement |
| ☒ | ☐ | A statement on whether measurements were taken from distinct samples or whether the same sample was measured repeatedly |
| ☐ | ☒ | The statistical test(s) used AND whether they are one- or two-sided<br>*Only common tests should be described solely by name; describe more complex techniques in the Methods section.* |
| ☒ | ☐ | A description of all covariates tested |
| ☐ | ☒ | A description of any assumptions or corrections, such as tests of normality and adjustment for multiple comparisons |
| ☐ | ☒ | A full description of the statistical parameters including central tendency (e.g. means) or other basic estimates (e.g. regression coefficient) AND variation (e.g. standard deviation) or associated estimates of uncertainty (e.g. confidence intervals) |
| ☒ | ☐ | For null hypothesis testing, the test statistic (e.g. *F*, *t*, *r*) with confidence intervals, effect sizes, degrees of freedom and *P* value noted<br>*Give P values as exact values whenever suitable.* |
| ☒ | ☐ | For Bayesian analysis, information on the choice of priors and Markov chain Monte Carlo settings |
| ☒ | ☐ | For hierarchical and complex designs, identification of the appropriate level for tests and full reporting of outcomes |
| ☐ | ☒ | Estimates of effect sizes (e.g. Cohen's *d*, Pearson's *r*), indicating how they were calculated |

*Our web collection on statistics for biologists contains articles on many of the points above.*

## Software and code

Policy information about availability of computer code

| | |
|---|---|
| Data collection | No software were used |
| Data analysis | We used R version 4.3 for analyzing the data |

For manuscripts utilizing custom algorithms or software that are central to the research but not yet described in published literature, software must be made available to editors and reviewers. We strongly encourage code deposition in a community repository (e.g. GitHub). See the Nature Portfolio guidelines for submitting code & software for further information.

## Data

Policy information about availability of data

All manuscripts must include a data availability statement. This statement should provide the following information, where applicable:

- Accession codes, unique identifiers, or web links for publicly available datasets
- A description of any restrictions on data availability
- For clinical datasets or third party data, please ensure that the statement adheres to our policy

The scRNA-seq data for breast cancer and normal patients are publicly available through the Gene Expression Omnibus under accession number GSE176078 and GSE161529, respectively. The ATAC-seq data for BC cell lines used in this study are publicly available through the Gene Expression Omnibus under accession number GSE114964, GSE174152, GSE89013 and GSE121370.

# Research involving human participants, their data, or biological material

Policy information about studies with human participants or human data. See also policy information about sex, gender (identity/presentation), and sexual orientation and race, ethnicity and racism.

| | |
|---|---|
| Reporting on sex and gender | The data was from breast cancer and accordingly only female were included. No gender differences were included. |
| Reporting on race, ethnicity, or other socially relevant groupings | Our research was a meta-analysis and we collected the breast cancer data from previously published papers. Hence, we did not include any race, ethnicity or other social relevant. |
| Population characteristics | The meta-data of the patients included in our research can be found in the original papers. |
| Recruitment | Our research was a meta-analysis of previously published articles. Hence, we did not recruit any patients. |
| Ethics oversight | Our research was a meta-analysis of previously published articles. Hence, ethical approvals can be found in the original publications. |

Note that full information on the approval of the study protocol must also be provided in the manuscript.

# Field-specific reporting

Please select the one below that is the best fit for your research. If you are not sure, read the appropriate sections before making your selection.

☒ Life sciences ☐ Behavioural & social sciences ☐ Ecological, evolutionary & environmental sciences

For a reference copy of the document with all sections, see nature.com/documents/nr-reporting-summary-flat.pdf

# Life sciences study design

All studies must disclose on these points even when the disclosure is negative.

| | |
|---|---|
| Sample size | Single cell RNA sequencing data from 33 BC patients, 20 BC and 13 normal, were extracted from previously published papers. Overall more than 15000 epithelial cells were studied. Our approach was to include all available data which meets the inclusion criteria (1-BC patient 2-hetorgenity between patients 3- having inferCNV calculation for the BC patients) |
| Data exclusions | The data from patients which their epithelial cells could not be distinguished to normal and cancer states were excluded |
| Replication | The scRNAseq data were supported using ATAC-seq. Our findings for the drugs which positively affect breast cancer progression, have several supports in previously published papers. |
| Randomization | In this study, we would like to see the differences between different breast cancer subtypes comparing to normal. Hence, randomization is not applicable in our study. |
| Blinding | Our research was a meta-analysis of previously published articles. |

# Reporting for specific materials, systems and methods

We require information from authors about some types of materials, experimental systems and methods used in many studies. Here, indicate whether each material, system or method listed is relevant to your study. If you are not sure if a list item applies to your research, read the appropriate section before selecting a response.

## Materials & experimental systems

| n/a | Involved in the study |
|---|---|
| ☒ | ☐ Antibodies |
| ☒ | ☐ Eukaryotic cell lines |
| ☒ | ☐ Palaeontology and archaeology |
| ☒ | ☐ Animals and other organisms |
| ☒ | ☐ Clinical data |
| ☒ | ☐ Dual use research of concern |
| ☒ | ☐ Plants |

## Methods

| n/a | Involved in the study |
|---|---|
| ☒ | ☐ ChIP-seq |
| ☒ | ☐ Flow cytometry |
| ☒ | ☐ MRI-based neuroimaging |

## Plants

| | |
|---|---|
| Seed stocks | *Report on the source of all seed stocks or other plant material used. If applicable, state the seed stock centre and catalogue number. If plant specimens were collected from the field, describe the collection location, date and sampling procedures.* |
| Novel plant genotypes | *Describe the methods by which all novel plant genotypes were produced. This includes those generated by transgenic approaches, gene editing, chemical/radiation-based mutagenesis and hybridization. For transgenic lines, describe the transformation method, the number of independent lines analyzed and the generation upon which experiments were performed. For gene-edited lines, describe the editor used, the endogenous sequence targeted for editing, the targeting guide RNA sequence (if applicable) and how the editor was applied.* |
| Authentication | *Describe any authentication procedures for each seed stock used or novel genotype generated. Describe any experiments used to assess the effect of a mutation and, where applicable, how potential secondary effects (e.g. second site T-DNA insertions, mosiacism, off-target gene editing) were examined.* |
