# Supplementary material for: Drug repositioning for immunotherapy in breast cancer using single-cell analysis
Source: NPJ Syst Biol Appl. 2024 Apr 8;10:37. doi: 10.1038/s41540-024-00359-z (PMC11001976; doi:10.1038/s41540-024-00359-z)
Supplement: Supplementary file 2 — Supplementary Files [file 41540_2024_359_MOESM2_ESM.pdf]

Supplementary Figures

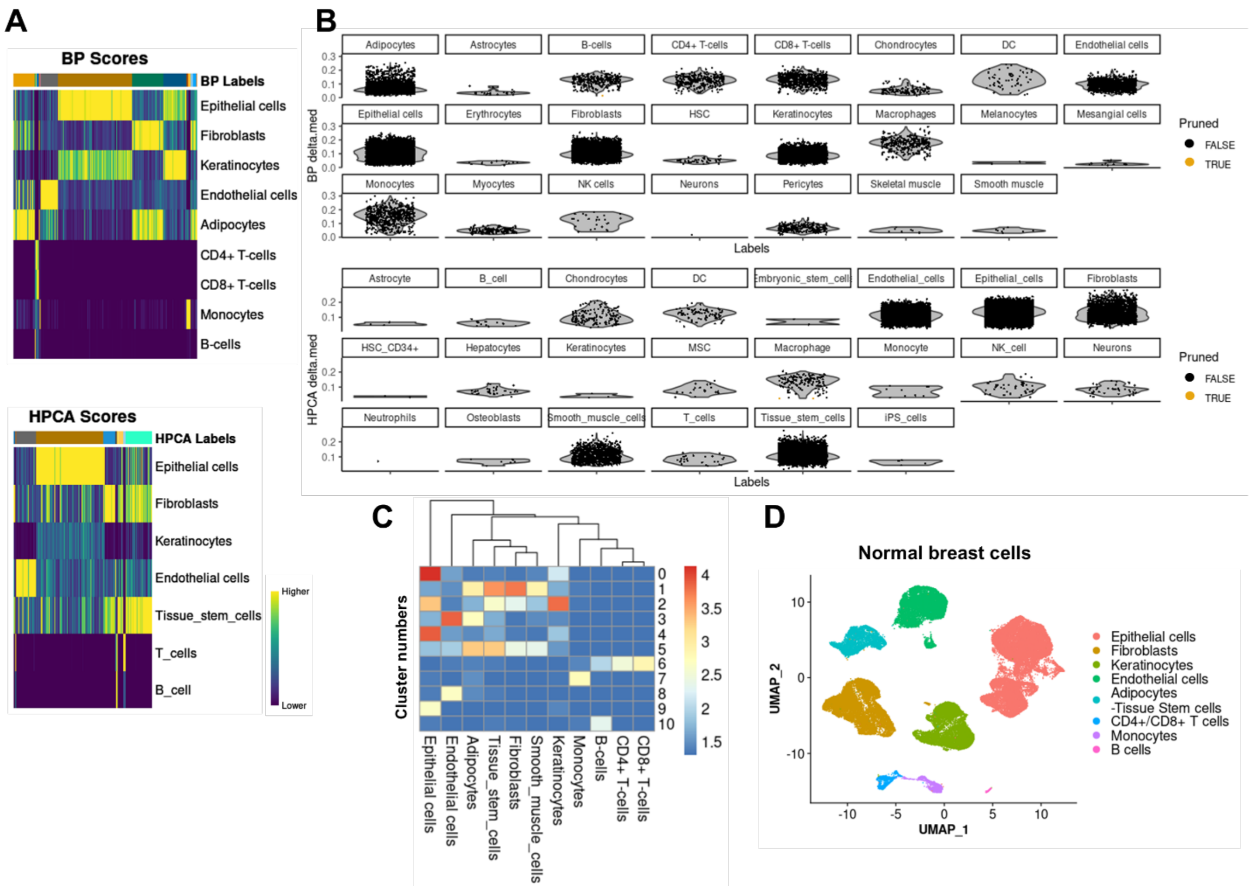

Supplementary Figure 1. Using a combination of previously labeled datasets as a reference, automatic annotation can define cell types in scRNA-seq analysis. A) Heatmaps of a nested matrix of per-cell scores showing correlation-based scores, prior to any fine-tuning, for each cell (rows), reference label color (columns) and reference label assignment as row names. **B)** Measuring the delta

values for each cell in Panel A, i.e., the difference between the score for the assigned label and the median score across all labels for each cell. Outliers are shown in yellow. **C)** Investigating the distribution of assigned cell labels in Panel B to the clusters defined in Panel A. Higher values indicate stronger concordance between two methods. Row numbers represent the cluster number in panel A. **D**) Assignment of labels deduced in Panel A to defined clusters.

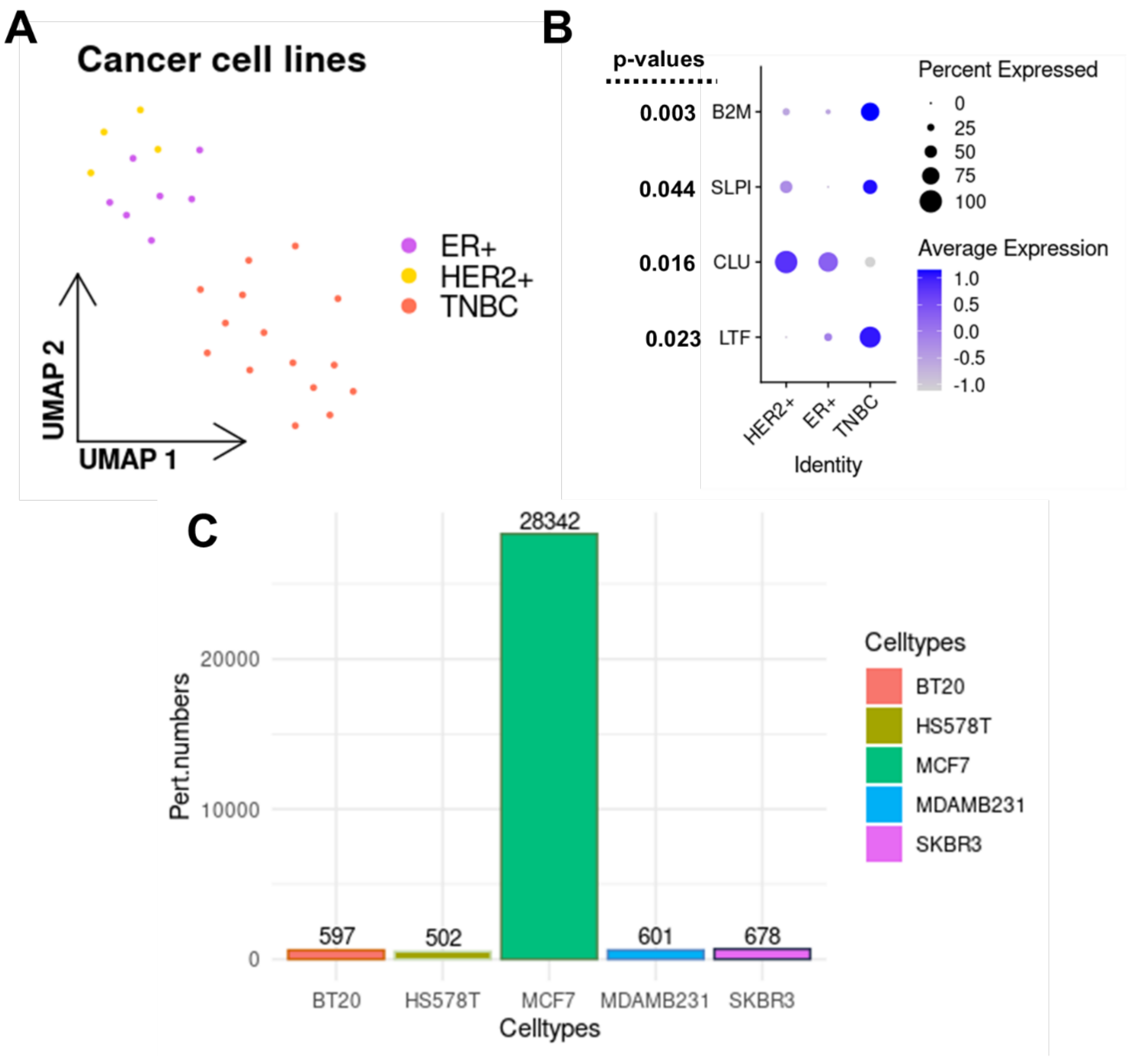

A
Cancer cell lines
ER+
HER2+
TNBC
UMAP 2
UMAP 1
B
p-values
0.003 B2M
0.044 SLPI
0.016 CLU
0.023 LTF
HER2+ ER+ TNBC
Identity
Percent Expressed
0
25
50
75
100
Average Expression
1.0
0.5
0.0
-0.5
-1.0
C
Pert.numbers
0
10000
20000
597
502
28342
601
678
BT20 HS578T MCF7 MDAMB231 SKBR3
Celltypes
Celltypes
BT20
HS578T
MCF7
MDAMB231
SKBR3

Supplementary Figure 2. Cancer cell line data analysis. A) UMAP visualization of proteomics profiles of different BC subtypes. **B)** Differential expression of selected AMPs which were available in the proteomics profiles of different BC cell lines **C)** Number of available perturbagens for all BC cell lines in LINCS L1000 database.

Supplementary Tables

Supplementary Table 1. The therapeutic peptides metadata which were considered in this study

UDAMP ID	Gene name	UniProt ID	Protein Name	Length	Source database ID	GI number	PDB ID	Antimicrobial activity	Gram nature	Type of validation
UDAMP1	FAU	P62861	40S ribosomal protein S30	59	AP02096	Not available	4V6X	Antibacterial	Gram positive,Gram negative	Experimentally Validated
UDAMP2	ADM	P35318	Adrenomedullin	52	CAMPSQ3270	Not available	2L7S	Antibacterial	Gram positive, Gram negative	Experimentally Validated
UDAMP3	SERPINA1	P01009	Alpha-1-antitrypsin	20	dbAMP_00153	Not available	6ROD	Antimicrobial	Not available	Predicted
UDAMP4	SNCA	P37840	Alpha-synuclein	140	AP02851	Not available	Not available	Antibacterial, Antifungal	Gram positive,Gram negative	Experimentally Validated
UDAMP5	APP	P05067	Amyloid-beta precursor protein	40	CAMPSQ3418	Not available	1AML	Antibacterial, Antifungal	Gram positive, Gram negative	Experimentally Validated
UDAMP6	ANG	P03950	Angiogenin	125	CAMPSQ3726	Not available	1B1I	Antibacterial, Antifungal	Gram positive	Experimentally Validated
UDAMP7	SLPI	P03973	Antileukoproteinase	107	CAMPSQ2165	Not available	Not available	Antibacterial, Antifungal, Antiviral	Gram positive, Gram negative	Predicted
UDAMP8	ATP5F1B	P06576	ATP synthase subunit beta, mitochondrial	13	DBAASP13431	Not available	Not available	Antibacterial,	Gram positive,Gram negative	Experimentally Validated
UDAMP9	AZU1	P20160	Azurocidin	222	CAMPSQ1515	416746	1A7S,1AE5,1FY1, 1FY3	Antibacterial	Gram negative	Experimentally Validated
UDAMP10	BPI	P17213	Bactericidal permeability-increasing protein	456	CAMPSQ2157	Not available	1BP1,1EWF	Antibacterial	Gram negative	Experimentally Validated
UDAMP11	B2M	P61769	Beta-2-microglobulin	99	AP02343	Not available	2YXF, 2D4F	Antibacterial	Gram positive,Gram negative	Experimentally Validated
UDAMP12	CSN2	P05814	Beta-casein	14	AP02774	Not available	Not available	Antibacterial	Gram positive,Gram negative	Experimentally validated
UDAMP13	DEFB105B	B2RU30	Beta-defensin	78	CAMPSQ4980	187957636	Not available	Antimicrobial	Not available	Predicted (Based on signature)
UDAMP14	DEFB117	Q30KQ3	Beta-defensin	42	CAMPSQ7591	Not available	6MJV	Antimicrobial	Not available	Predicted
UDAMP15	DEFB1	P60022	Beta-defensin 1	36	CAMPSQ1601	38503374	1E4S,1IJU,1IJV, 1KJ5,2	Antibacterial	Not available	Predicted

							NLB,2NLC,2NLD,2NLE,2NLF,2NLG,2NLH,2NLP,2NLQ,2NLS,2PLZ			
UDAMP16	DEFB104A	Q8WTQ1	Beta-defensin 104	49	CAMPSQ2843	Not available	5KI9	Antibacterial	Gram positive, Gram negative	Experimentally Validated
UDAMP17	DEFB105A	Q8NG35	Beta-defensin 105	51	CAMPSQ1447	22748617	Not available	Antimicrobial	Not available	Predicted
UDAMP18	DEFB106A	Q8N104	Beta-defensin 106	45	CAMPSQ1375	103485143	2LWL	Antimicrobial	Not available	Experimentally Validated
UDAMP19	DEFB107A	Q8IZN7	Beta-defensin 107	44	CAMPSQ1374	103485147	Not available	Antimicrobial	Not available	Predicted
UDAMP20	DEFB108A	A8MXU0	Beta-defensin 108A	51	CAMPSQ1978	190359191	Not available	Antibacterial	Not available	Predicted
UDAMP21	DEFB112	Q30KQ8	Beta-defensin 112	113	CAMPSQ4359	Not available	Not available	Antibacterial	Not available	Predicted
UDAMP22	DEFB118	Q96PH6	Beta-defensin 118	43	CAMPSQ1934	23813949	Not available	Antibacterial	Not available	Predicted
UDAMP23	DEFB119	Q8N690	Beta-defensin 119	63	CAMPSQ1931	41713338	Not available	Antibacterial	Not available	Predicted
UDAMP24	DEFB125	Q8N687	Beta-defensin 125	47	CAMPSQ1905	152112501	Not available	Antibacterial	Not available	Predicted
UDAMP25	DEFB126	Q9BYW3	Beta-defensin 126	42	CAMPSQ2740	Not available	Not available	Antibacterial	Gram negative	Experimentally Validated
UDAMP26	DEFB127	Q9H1M4	Beta-defensin 127	44	CAMPSQ2741	Not available	Not available	Antibacterial	Gram negative	Experimentally Validated
UDAMP27	DEFB128	Q7Z7B8	Beta-defensin 128	37	CAMPSQ3159	Not available	Not available	Antimicrobial	Not available	Experimentally Validated
UDAMP28	DEFB130	Q30KJ9	Beta-defensin 130	57	dbAMP_04245	Not available	Not available	Antibacterial, Antiparasitic	Not available	Predicted
UDAMP29	DEFB4A	O15263	Beta-defensin 4A	41	CAMPSQ486	4826692	1E4Q,1FD3,1FD4,1FQQ	Antibacterial	Gram positive, Gram negative	Experimentally Validated
UDAMP30	DEFB103A	P81534	Beta-defensin103	45	CAMPSQ657	9756284	1KJ6	Antibacterial, Antifungal	Gram positive, Gram negative	Experimentally Validated
UDAMP31	DEFB108B	Q8NET1	Beta-defensin108B	51	CAMPSQ1902	37154739	Not available	Antibacterial	Not available	Predicted
UDAMP32	DEFB109B	Q30KR1	Beta-defensin109	65	CAMPSQ2100	84028868	Not available	Antibacterial	Not available	Predicted
UDAMP33	DEFB110	Q30KQ9	Beta-defensin111	48	CAMPSQ2097	84028871	Not available	Antibacterial	Not available	Predicted
UDAMP34	DEFB113	Q30KQ7	Beta-defensin113	66	CAMPSQ2095	84028875	Not available	Antibacterial	Not available	Predicted
UDAMP35	DEFB114	Q30KQ6	Beta-defensin114	43	CAMPSQ2077	85540943	Not available	Antibacterial	Not available	Experimentally Validated
UDAMP36	DEFB115	Q30KQ5	Beta-defensin115	61	CAMPSQ2093	84028877	Not available	Antibacterial	Not available	Predicted

UDAMP37	DEFB116	Q30KQ4	Beta-defensin116	79	CAMPSQ2092	84028878	Not available	Antibacterial	Not available	Predicted
UDAMP38	DEFB121	Q5J5C9	Beta-defensin121	61	CAMPSQ2111	61212937	Not available	Antibacterial	Not available	Predicted
UDAMP39	DEFB123	Q8N688	Beta-defensin123	47	CAMPSQ1933	26392719	Not available	Antibacterial	Not available	Predicted
UDAMP40	DEFB124	Q8NES8	Beta-defensin124	49	CAMPSQ2101	84028202	Not available	Antibacterial	Not available	Predicted
UDAMP41	DEFB128	Q7Z7B8	Beta-defensin128	75	CAMPSQ2108	61212983	Not available	Antibacterial	Not available	Predicted
UDAMP42	DEFB129	Q9H1M3	Beta-defensin129	164	CAMPSQ2113	26392781	Not available	Antibacterial	Not available	Predicted
UDAMP43	DEFB131A	P59861	Beta-defensin131A	48	CAMPSQ1932	37076854	Not available	Antibacterial	Not available	Predicted
UDAMP44	DEFB132	Q7Z7B7	Beta-defensin132	73	CAMPSQ2112	61212742	Not available	Antibacterial	Not available	Predicted
UDAMP45	DEFB133	Q30KQ1	Beta-defensin133	38	CAMPSQ2084	84028894	Not available	Antibacterial	Not available	Predicted
UDAMP46	DEFB134	Q4QY38	Beta-defensin134	47	CAMPSQ2107	74762871	Not available	Antibacterial	Not available	Predicted
UDAMP47	DEFB135	Q30KP9	Beta-defensin135	53	CAMPSQ2081	84028897	Not available	Antibacterial	Not available	Predicted
UDAMP48	DEFB136	Q30KP8	Beta-defensin136	57	CAMPSQ2079	84028899	Not available	Antibacterial	Not available	Predicted
UDAMP49	PRG2	P13727	Bone marrow proteoglycan	206	CAMPSQ2035	119239	1H8U,2BRS	Antiparasitic	Not available	Experimentally Validated
UDAMP50	BST2	Q10589	Bone marrow stromal antigen 2	121	dbAMP_00209	Not available	3MQB&&3MQC, 3NWH	Antiviral	Not available	Experimentally validated
UDAMP51	BPIFA1	Q9NP55	BPI fold-containing family A member 1 precursor	255	CAMPSQ2674	342307069	Not available	Antibacterial	Gram negative	Experimentally Validated
UDAMP52	BPIFA2	Q96DR5	BPI fold-containing family A member 2 (Parotid secretory protein) (PSP) (Short palate, lung and nasal epithelium carcinoma-associated protein 2)	231	CAMPSQ7528	Not available	Not available	Antibacterial	Gram negative	Experimentally Validated
UDAMP53	BPIFB3	P59826	BPI fold-containing family B member 3	458	CAMPSQ1415	73620973	Not available	Antimicrobial	Not available	Predicted
UDAMP54	BPIFB4	P59827	BPI fold-containing family B member 4	575	CAMPSQ1416	34395623	Not available	Antimicrobial	Not available	Predicted
UDAMP55	CALCA	P06881	Calcitonin gene-related peptide 1	37	CAMPSQ3267	Not available	Not available	Antibacterial, Antifungal	Gram positive, Gram negative	Experimentally Validated

UDAMP56	CAMP	P49913	Cathelicidin antimicrobial peptide	67	CAMPSQ1409	39753970	4EYC	Antimicrobial	Not available	Predicted
UDAMP57	CTSG	P08311	Cathepsin G	235	CAMPSQ4485	Not available	1AU8, 1CGH, 1KYN, 1T32	Antibacterial	Gram negative	Experimentally Validated
UDAMP58	CCL1	P22362	C-C motif chemokine 1	73	CAMPSQ3744	Not available	1EL0	Antibacterial	Gram positive, Gram negative	Experimentally Validated
UDAMP59	CCL13	Q99616	C-C motif chemokine 13	75	CAMPSQ3747	Not available	2RA4	Antibacterial	Gram negative	Experimentally Validated
UDAMP60	CCL17	Q92583	C-C motif chemokine 17	71	CAMPSQ3748	Not available	Not available	Antibacterial	Gram positive, Gram negative	Experimentally Validated
UDAMP61	CCL18	P55774	C-C motif chemokine 18	69	CAMPSQ3749	Not available	4MHE	Antibacterial	Gram negative	Experimentally Validated
UDAMP62	CCL19	Q99731	C-C motif chemokine 19	77	CAMPSQ3750	Not available	2MP1	Antibacterial	Gram positive, Gram negative	Experimentally Validated
UDAMP63	CCL20	P78556	C-C motif chemokine 20	69	CAMPSQ3734	Not available	1M8A	Antibacterial	Gram positive, Gram negative	Experimentally Validated
UDAMP64	CCL21	O00585	C-C motif chemokine 21	111	CAMPSQ3752	Not available	2L4N	Antibacterial	Gram positive, Gram negative	Experimentally Validated
UDAMP65	CCL22	O00626	C-C motif chemokine 22	69	CAMPSQ3753	Not available	Not available	Antibacterial	Gram positive, Gram negative	Experimentally Validated
UDAMP66	CCL24	O00175	C-C motif chemokine 24	78	dbAMP_11958	Not available	1EIG	Antibacterial	Gram positive,Gram negative	Experimentally validated
UDAMP67	CCL25	O15444	C-C motif chemokine 25	127	CAMPSQ3751	Not available	Not available	Antibacterial	Gram positive, Gram negative	Experimentally Validated
UDAMP68	CCL27	Q9Y4X3	C-C motif chemokine 27	22	dbAMP_02391	Not available	2KUM	Antibacterial	Not available	Experimentally validated
UDAMP69	CCL28	Q9NRJ3	C-C motif chemokine 28	127	CAMPSQ3829	Not available	6CWS	Antibacterial	Gram positive, Gram negative	Experimentally Validated
UDAMP70	CCL4	P13236	C-C motif chemokine 4	69	dbAMP_00453	Not available	1EL0	Antiviral	Not available	Experimentally validated
UDAMP71	CCL8	P80075	C-C motif chemokine 8	75	CAMPSQ3745	Not available	Not available	Antibacterial	Gram negative	Experimentally Validated
UDAMP72	CHGA	P10645	Chromogranin-A	21	CAMPSQ2919	Not available	6R2X	Antibacterial, Antifungal	Gram positive, Gram negative	Experimentally Validated
UDAMP73	CLU	P10909	Clusterin	449	28120874	Not available	Not available	Antimicrobial	Not available	Not available
UDAMP74	CXCL10	P02778	C-X-C motif chemokine 10	77	CAMPSQ3739	Not available	1O80	Antibacterial	Gram positive, Gram negative	Experimentally Validated

UDAMP75	CXCL11	O14625	C-X-C motif chemokine 11	73	CAMPSQ3740	Not available	1RJT	Antibacterial	Gram positive, Gram negative	Experimentally Validated
UDAMP76	CXCL13	O43927	C-X-C motif chemokine 13	87	dbAMP_11826	Not available	4ZAI	Antibacterial	Gram positive,Gram negative	Experimentally validated
UDAMP77	CXCL14	O95715	C-X-C motif chemokine 14	77	CAMPSQ3194	Not available	2HDL	Antibacterial, Antifungal	Gram positive, Gram negative	Experimentally Validated
UDAMP78	CXCL2	P19875	C-X-C motif chemokine 2	73	CAMPSQ3736	Not available	3N52	Antibacterial	Gram positive, Gram negative	Experimentally Validated
UDAMP79	CXCL3	P19876	C-X-C motif chemokine 3	73	CAMPSQ3737	Not available	Not available	Antibacterial	Gram positive, Gram negative	Experimentally Validated
UDAMP80	CXCL6	P80162	C-X-C motif chemokine 6	77	CAMPSQ3828	Not available	Not available	Antibacterial, Antiparasitic	Gram positive, Gram negative	Experimentally Validated
UDAMP81	CXCL9	Q07325	C-X-C motif chemokine 9	103	CAMPSQ3738	Not available	Not available	Antibacterial	Gram positive, Gram negative	Experimentally Validated
UDAMP82	CST11	Q9H112	Cystatin-11	112	CAMPSQ1115	76803548	Not available	Antibacterial	Gram negative	Experimentally Validated
UDAMP83	CST5	P28325	Cystatin-D	142	21323710	Not available	1RN7	Antimicrobial	Not available	Not available
UDAMP84	CST4	P01036	Cystatin-S	121	21323710	Not available	Not available	Antimicrobial	Not available	Not available
UDAMP85	CST2	P09228	Cystatin-SA	141	21323710	Not available	Not available	Antimicrobial	Not available	Not available
UDAMP86	CST1	P01037	Cystatin-SN	141	21323710	Not available	Not available	Antimicrobial	Not available	Not available
UDAMP87	DEFA3	Q6EZE9	Defensin, alpha 3, neutrophil-specific	94	CAMPSQ6714	Not available	Not available	Antimicrobial	Not available	Predicted (Based on signature)
UDAMP88	DEFA5	Q01523	Defensin-5	32	CAMPSQ730	109156995	1ZMP,3I5W,4E82, 4E83,4E86	Antibacterial, Antifungal, Antiviral	Gram positive, Gram negative	Experimentally Validated
UDAMP89	DEFA6	Q01524	Defensin-6	32	CAMPSQ755	109156999	3QTE	Antibacterial	Gram positive	Experimentally Validated
UDAMP90	DMBT1	Q9UGM3	Deleted in malignant brain tumors 1 protein	2413	21614203	Not available	6SA4	Antimicrobial	Not available	Not available
UDAMP91	DCD	P81605	Dermcidin	47	CAMPSQ238	20141302	2KSG,2YMK	Antibacterial, Antifungal	Gram positive, Gram negative	Experimentally Validated
UDAMP92	APOBEC3G	Q9HC16	DNA dC->dU-editing enzyme APOBEC-3G	189	dbAMP_03919	Not available	3IQS&&3E1U	Antiviral	Not available	Experimentally validated
UDAMP93	PI3	P19957	Elafin	57	CAMPSQ1144	Not available	2REL	Antibacterial	Gram positive, Gram negative	Experimentally Validated

UDAMP94	RNASE3	P12724	Eosinophil cationic protein	133	CAMPSQ1961	147744558	1DYT,1H1H,1QMT, 2KB5,4A2O,4A2Y	Antibacterial	Not available	Experimentally Validated
UDAMP95	CCL11	P51671	Eotaxin	74	CAMPSQ3746	Not available	2EOT	Antibacterial	Gram positive, Gram negative	Experimentally Validated
UDAMP96	LACRT	Q9GZZ8	Extracellular glycoprotein lacritin	138	24942736	Not available	Not available	Antimicrobial	Not available	Not available
UDAMP97	FGA	P02671	Fibrinogen alpha chain	16	DBAASP12111	Not available	3H32,3GHG	Antibacterial, Antifungal	Gram positive,Gram negative	Experimentally Validated
UDAMP98	FLG2	Q5D862	Filaggrin-2	70	AP02602	Not available	Not available	Antibacterial	Gram negative	Experimentally Validated
UDAMP99	FURIN	P09958	Furin	26	AP03044	Not available	6A8Y	Antibacterial,	Gram positive,Gram negative	Experimentally validated
UDAMP100	GALP	Q9UBC7-2	Galanin-like peptide	25	CAMPSQ3827	Not available	Not available	Antibacterial	Gram negative	Experimentally Validated
UDAMP101	LGALS3	P17931	Galectin-3	249	CAMPSQ2163	Not available	1A3K,1KJL,1KJR, 2NMN, 2NMO,2NN8,2XG3, 3AYA, 3AYC,3AYD,3AYE, 3T1L, 3T1M,3ZSJ,3ZSK, 3ZSL,3ZSM	Antibacterial, Antifungal	Not available	Experimentally Validated
UDAMP102	GAPDH	P04406	Glyceraldehyde-3-phosphate dehydrogenase	31	CAMPSQ3694	Not available	6YND, 6YNF	Antibacterial, Antifungal	Gram negative	Experimentally Validated
UDAMP103	GNLY	P22749	Granulysin	83	CAMPSQ3084	Not available	1L9L	Antibacterial, Antifungal	Gram positive, Gram negative	Experimentally Validated
UDAMP104	CXCL1	P09341	Growth-regulated alpha protein	73	CAMPSQ3735	Not available	1MSH	Antibacterial	Gram positive, Gram negative	Experimentally Validated
UDAMP105	GBP1	P32455	Guanylate-binding protein 1	589	CAMPSQ7544	Not available	6K1Z	Antiviral	Not available	Experimentally Validated
UDAMP106	GBP2	P32456	Guanylate-binding protein 2	588	CAMPSQ7545	Not available	Not available	Antiviral	Not available	Predicted
UDAMP107	HP	P00738	Haptoglobin beta chain	245	CAMPSQ4511	Not available	Not available	Antibacterial,	Not available	Experimentally Validated
UDAMP108	SERPIND1	P05546	Heparin cofactor 2	91	dbAMP_12372	Not available	Not available	Antibacterial, Antifungal	Gram positive,Gram negative	Experimentally Validated
UDAMP109	HAMP	P81172	Hepcidin	25	CAMPSQ983	159162673	1M4E,1M4F,2KEF, 3H0T	Antibacterial, Antifungal	Gram positive, Gram negative	Experimentally Validated

UDAMP110	HAMP	P81172	Hepicidin-20	20	DBAASP2043	Not available	2KEF	Antibacterial, Antifungal	Gram positive,Gram negative	Experimentally Validated
UDAMP111	HTN1	P15515	Histatin-1	38	CAMPSQ738	123134	Not available	Antifungal	Not available	Experimentally Validated
UDAMP112	HTN3	P15516	Histatin-3	32	CAMPSQ103	123143	Not available	Antifungal	Not available	Experimentally Validated
UDAMP113	HTN3	P15516	Histatin-5	24	CAMPSQ899	Not available	Not available	Antifungal	Not available	Experimentally Validated
UDAMP114	HTN3	P15516	Histatin-8	12	CAMPSQ2790	Not available	Not available	Antifungal	Not available	Experimentally Validated
UDAMP115	HRG	P04196	Histidine-rich glycoprotein	21	CAMPSQ3284	Not available	Not available	Antifungal	Not available	Experimentally Validated
UDAMP116	H2AFJ	Q9BTM1	Histone H2A.J	39	AP00307	Not available	6KVD	Antibacterial, Antifungal	Gram positive, Gram negative	Experimentally Validated
UDAMP117	HIST1H2BC	P62807	Histone H2B type 1-C/E/F/G/I	125	CAMPSQ3885	Not available	Not available	Antibacterial	Gram negative	Experimentally Validated
UDAMP118	HRNR	Q86YZ3	Hornerin	12	AP03020	Not available	Not available	Antibacterial	Gram negative	Experimentally validated
UDAMP119	IFNA2	P01563	Interferon alpha-2	165	dbAMP_00792	Not available	Not available	Antiviral	Not available	Experimentally validated
UDAMP120	IFNL3	Q8IZI9	Interferon lambda-3	196	dbAMP_09451	Not available	3HHC	Antiviral	Not available	Experimentally validated
UDAMP121	MX1	P20591	Interferon-induced GTP-binding protein Mx1	271	dbAMP_01296	Not available	3LJB	Antiviral	Not available	Experimentally validated
UDAMP122	IFIH1	Q9BYX4	Interferon-induced helicase C domain-containing protein 1	216	dbAMP_11175	Not available	3B6E	Antiviral	Not available	Experimentally validated
UDAMP123	ISG20	Q96AZ6	Interferon-stimulated gene 20 kDa protein	189	dbAMP_06226	Not available	1WLJ	Antiviral	Not available	Experimentally validated
UDAMP124	IL26	Q9NPH9	Interleukin-26	171	AP03086	Not available	Not available	Antibacterial	Gram negative	Experimentally Validated
UDAMP125	CXCL8	P10145	Interleukin-8	77	DBAASP8505	Not available	6N2U	Antibacterial	Gram positive,Gram negative	Experimentally validated
UDAMP126	IAPP	P10997	Islet amyloid polypeptide	37	CAMPSQ3834	Not available	2L86	Antimicrobial	Not available	Experimentally Validated
UDAMP127	KLK5	Q9Y337	Kallikrein-5	227	CAMPSQ1445	117306176	2PSX,2PSY	Antimicrobial	Not available	Predicted

UDAMP128	KRT6C	P48668	Keratin, type II cytoskeletal 6C	19	AP02231	Not available	Not available	Antibacterial	Gram negative	Experimentally Validated
UDAMP129	KNG1	P01042	Kininogen-1	10	CAMPSQ3263	Not available	Not available	Antibacterial, Antifungal	Not available	Experimentally Validated
UDAMP130	LTF	P02788	Lactotransferrin	49	CAMPSQ2882	Not available	1Z6V	Antimicrobial	Not available	Experimentally Validated
UDAMP131	LCN1	P31025	Lipocalin-1	157	CAMPSQ2164	Not available	1XKI,3EYC	Antibacterial	Gram negative	Predicted
UDAMP132	LEAP2	Q969E1	Liver-expressed antimicrobial peptide 2	40	CAMPSQ739	20138667	2L1Q	Antibacterial	Not available	Experimentally Validated
UDAMP133	XCL1	P47992	Lymphotactin	93	CAMPSQ3743	Not available	1J8I	Antibacterial	Gram positive, Gram negative	Experimentally Validated
UDAMP134	LYZ	P61626	Lysozyme C	130	CAMPSQ4552	Not available	133L, 134L, 1B5U, 1B5V, 1B5W, 1B5X, 1B5Y, 1B5Z, 1B7L, 1B7M, 1B7N, 1B7O, 1B7P, 1B7Q, 1B7R, 1B7S, 1BB3, 1BB4, 1BB5, 1C43, 1C45, 1C46, 1C7P, 1CJ6, 1CJ7, 1CJ8, 1CJ9, 1CKC, 1CKD, 1CKF, 1CKG, 1CKH, 1D6P, 1D6Q, 1DI3, 1DI4, 1DI5, 1EQ4, 1EQ5, 1EQE, 1GAY, 1GAZ, 1GB	Antibacterial	Not available	Predicted
UDAMP135	SCGB2A1	O75556	Mammaglobin-B	77	16395610	Not available	Not available	Antimicrobial	Not available	Not available
UDAMP136	POMC	P01189	Melanocyte-stimulating hormone alpha(Pro-opiomelanocortin)	13	CAMPSQ2779	Not available	Not available	Antibacterial, Antifungal	Gram positive	Experimentally Validated
UDAMP137	MUC7	Q8TAX7	Mucin-7	20	CAMPSQ2789	Not available	Not available	Antibacterial, Antifungal	Gram positive, Gram negative	Experimentally Validated
UDAMP138	NPPB	P16860	Natriuretic peptides B	32	L03A000342	Not available	Not available	Antimicrobial	Not available	Experimentally Validated

UDAMP139	NPY	P01303	Neuropeptide Y	36	CAMPSQ3265	Not available	1RON	Antifungal	Not available	Experimentally Validated
UDAMP140	VGF	O15240	Neurosecretory protein VGF	30	CAMPSQ8137	Not available	Not available	Antibacterial	Gram positive	Experimentally Validated
UDAMP141	NTS	P30990	Neurotensin	13	dbAMP_01461	Not available	2LNE	Antimicrobial	Not available	Experimentally validated
UDAMP142	DEFA1	P59665	Neutrophil defensin 1	29	CAMPSQ2738	Not available	3HJ2	Antibacterial	Gram positive, Gram negative	Experimentally Validated
UDAMP143	DEFA3	P59666	Neutrophil defensin 3	30	CAMPSQ345	30316323, 4503305	1DFN,1ZMH,1ZMI, 1ZMK,2PM4,2PM5	Antibacterial, Antifungal, Antiviral	Not available	Experimentally Validated
UDAMP144	DEFA4	P12838	Neutrophil defensin 4	33	CAMPSQ396	399352	1ZMM	Antibacterial, Antifungal	Gram positive, Gram negative	Experimentally Validated
UDAMP145	LCN2	P80188	Neutrophil gelatinase-associated lipocalin	179	dbAMP_03964	Not available	3TF6	Antibacterial	not available	Experimentally validated
UDAMP146	HMGN2	P05204	Non-histone chromosomal protein HMG-17	89	AP02230	Not available	Not available	Antibacterial, Antiviral, Antifungal	Gram negative	Experimentally Validated
UDAMP147	PYY	P10082	Peptide YY	36	DBAASP4190	Not available	2NA5	Antifungal	Not available	Experimentally Validated
UDAMP148	PGLYRP3	Q96LB9	Peptidoglycan recognition protein 3	255	CAMPSQ3864	Not available	1SK3,1SK4	Antibacterial	Gram negative	Experimentally Validated
UDAMP149	PGLYRP4	Q96LB8	Peptidoglycan recognition protein 4	255	CAMPSQ3863	Not available	Not available	Antibacterial	Gram negative	Experimentally Validated
UDAMP150	PRF1	P14222	Perforin-1	534	CAMPSQ2159	Not available	Not available	Antimicrobial	Not available	Experimentally Validated
UDAMP151	PLA2G2A	P14555	Phospholipase A2, membrane associated	124	AP02534	Not available	Not available	Antibacterial	Gram positive,Gram negative	Experimentally Validated
UDAMP152	PPBP	P02775	Platelet Basic protein	83	CAMPSQ3196	Not available	Not available	Antibacterial, Antifungal	Gram positive, Gram negative	Experimentally Validated
UDAMP153	PF4	P02776	Platelet factor 4	70	CAMPSQ3805	Not available	1F9Q	Antiparasitic	Not available	Experimentally Validated
UDAMP154	PIGR	P01833	Polymeric immunoglobulin receptor	746	29751532	Not available	6UE7_2	Antimicrobial	Not available	Not available

UDAMP155	PRR4	Q16378	Proline-rich protein 4	134	25141976	Not available	Not available	Antimicrobial	Not available	Not available
UDAMP156	TOR2A	Q8N2E6	Prosalusin	20	dbAMP_00230	Not available	Not available	Antibacterial	Gram positive	Experimentally validated
UDAMP157	TAC1	P20366	Protachykinin-1	11	CAMPSQ3262	Not available	Not available	Antibacterial, Antifungal	Gram positive	Experimentally Validated
UDAMP158	GPR15L	Q6UWK7	Protein GPR15L	57	CAMPSQ7536	Not available	Not available	Antibacterial, Antifungal, Antiviral	Gram positive	Experimentally Validated
UDAMP159	S100A12	P80511	Protein S100-A12	15	CAMPSQ846	2507565	1E8A,1GQM, 1ODB, 2WC8,2WCB, 2WCE,2WCF	Antibacterial, Antifungal	Gram negative	Experimentally Validated
UDAMP160	S100A8	P05109	Protein S100-A8	93	10771424	Not available	Not available	Antimicrobial	Not available	Not available
UDAMP161	S100A7	P31151	Protein S100-A7	101	CAMPSQ3731	Not available	2WND	Antibacterial	Gram negative	Experimentally Validated
UDAMP162	S100A9	P06702	Protein S100-A9	114	CAMPSQ2162	Not available	1IRJ	Antifungal	Not available	Experimentally Validated
UDAMP163	F2	P00734	Prothrombin	96	CAMPSQ3345	Not available	2GP9	Antibacterial, Antifungal	Gram positive, Gram negative	Experimentally Validated
UDAMP164	IQGAP2	Q13576	Ras GTPase-activating-like protein IQGAP2	23	dbAMP_01501	Not available	Not available	Antimicrobial	Not available	Experimentally validated
UDAMP165	ROMO1	P60602	Reactive oxygen species modulator 1	19	CAMPSQ3777	Not available	Not available	Antimicrobial	Not available	Experimentally Validated
UDAMP166	REG3A	Q06141	Regenerating islet-derived protein 3-alpha	149	CAMPSQ3730	Not available	2GO0	Antibacterial	Gram positive, Gram negative	Experimentally Validated
UDAMP167	RETN	Q9HD89	Resistin	92	AP03085	Not available	Not available	Antibacterial	Gram positive,Gram negative	Experimentally Validated
UDAMP168	RARRES2	Q99969	Retinoic acid receptor responder protein 2	20	CAMPSQ3833	Not available	Not available	Antibacterial	Gram positive, Gram negative	Experimentally Validated
UDAMP169	DLC1	Q96QB1	Rho GTPase-activating protein 7	29	dbAMP_10093	Not available	Not available	Antibacterial	Gram positive, Gram negative	Experimentally validated

UDAMP170	RNASE8	Q8TDE3	Ribonuclease 8	127	CAMPSQ3755	Not available	Not available	Antibacterial, Antifungal	Gram positive, Gram negative	Experimentally Validated
UDAMP171	RNASE6	Q93091	Ribonuclease K6	127	AP02425	Not available	4X09	Antibacterial	Gram positive,Gram negative	Experimentally Validated
UDAMP172	RNASE7	Q9H1E1	Ribonuclease7	128	CAMPSQ1105	20139729	2HKY	Antibacterial, Antifungal	Gram negative	Experimentally Validated
UDAMP173	SALV	Q86YR0	Salivary gland antimicrobial salvic	46	CAMPSQ793	Not available	Not available	Antibacterial	Gram positive, Gram negative	Experimentally Validated
UDAMP174	SCGB1D1	O95968	Secretoglobin family 1D member 1	69	30025099	Not available	Not available	Antimicrobial	Not available	Not available
UDAMP175	SEMG1	P04279	Semenogelin-1	29	CAMPSQ3220	Not available	Not available	Antimicrobial	Not available	Experimentally Validated
UDAMP176	SEMG2	Q02383	Semenogelin-2	29	CAMPSQ3221	Not available	Not available	Antibacterial	Gram positive, Gram negative	Experimentally Validated
UDAMP177	SPINK9	Q5DT21	Serine protease inhibitor Kazal-type 9	63	AP03038	Not available	not available	Antibacterial	Gram negative	Experimentally validated
UDAMP178	SPAG11B	Q08648	Sperm-associated antigen 11B	32	AP02888	Not available	Not available	Antibacterial	Gram negative	Experimentally Validated
UDAMP179	CXCL12	P48061	Stromal cell-derived factor 1	68	CAMPSQ3741	Not available	2KOL	Antibacterial	Gram positive, Gram negative	Experimentally Validated
UDAMP180	DCD	P81605	Survival-promoting peptide, Y-P30	30	DBAASP3199	Not available	Not available	Antibacterial, Antifungal	Gram positive,Gram negative	Experimentally validated
UDAMP181	TSLP	Q969D9	Thymic stromal lymphopoietin	63	AP02519	Not available	Not available	Antibacterial, Antifungal	Gram positive,Gram negative	Experimentally Validated
UDAMP182	SP1	P08047	Transcription factor Sp1	33	CAMPSQ4605	253723091	Not available	Antiviral	Not available	Experimentally Validated
UDAMP183	VIP	P01282	VIP peptides	28	CAMPSQ3268	Not available	2RRH	Antibacterial, Antifungal	Gram positive, Gram negative	Experimentally Validated
UDAMP184	WFDC12	Q8WWY7	WAP four-disulfide core domain protein12	88	CAMPSQ2029	24212610	Not available	Antibacterial	Not available	Predicted
UDAMP185	AZGP1	P25311	Zinc-alpha-2-glycoprotein	298	18677231	Not available	6R2U	Antimicrobial	Not available	Not available
UDAMP186	ZG16B	Q96DA0	Zymogen granule protein 16 homolog B	208	27256639	Not available	Not available	Antimicrobial	Not available	Not available

Supplementary Table 2. Maximum top 50 correlated genes with the selected AMPs.

Gene1	Gene2	cor	p	adj.p
**KRT6A**	DEFB1	0.307413152231623	0	0
**S100A9**	DEFB1	0.304178107142731	0	0
**S100A8**	DEFB1	0.30071627773622	0	0
**LTF**	PIP	0.300716560565217	0	0
**CLU**	S100A1	0.418482769577503	0	0
**SCGB2A2**	CLU	0.390698859642044	0	0
**CLU**	BCAM	0.380224907515248	0	0
**PIP**	CLU	0.35076795185137	0	0
**CLU**	CRABP2	0.346080849929166	0	0
**CLU**	MAOB	0.338800070503053	0	0
**CLU**	MT-ATP8	0.33299482563184	0	0
**CLU**	PYDC1	0.330291077341869	0	0
**SCGB1D2**	CLU	0.327359294982573	0	0
**CLU**	TRPS1	0.313945769718885	0	0
**CLU**	MLPH	0.311437620902444	0	0
**BTG2**	CLU	0.306564578535354	0	0
**CLU**	NME2	0.303080433169586	0	0
**CLU**	CRIP1	0.301645147059315	0	0
**SCGB2A1**	CLU	0.301308117302534	0	0
**CLU**	SHISA2	0.300863292611514	0	0
**SCGB2A1**	SCGB2A2	0.735297624824035	0	0
**SCGB2A1**	SCGB1D2	0.729446213146077	0	0
**SCGB2A1**	S100A1	0.532519960743452	0	0
**SCGB2A1**	PIP	0.473478727303245	0	0
**SCGB2A1**	MAOB	0.448721378517448	0	0
**SCGB2A1**	PYDC1	0.399278930875893	0	0

**SCGB2A1**	ZG16B	0.358641375501015	0	0
**SCGB2A1**	PPP1R1B	0.333411556929459	0	0
**SCGB2A1**	FOSB	0.327026816256927	0	0
**SCGB2A1**	CALML5	0.321999823289642	0	0
**SCGB2A1**	S100A14	0.317215469022331	0	0
**SCGB2A1**	CLDN3	0.307817724230864	0	0
**SCGB2A1**	CRABP2	0.306510571378824	0	0
**SCGB2A1**	CLU	0.301308117302534	0	0
**CRISP3**	SLPI	0.392656197661138	0	0
**SLPI**	TACSTD2	0.36229084586338	0	0
**SLPI**	HLA-A	0.336725613559164	0	0
**SLPI**	PHGR1	0.326840092459585	0	0
**SLPI**	B2M	0.320444888094031	0	0
**SLPI**	PDZK1IP1	0.309617810176879	0	0
**SLPI**	NUCB2	0.309223037503797	0	0
**SLPI**	IFI6	0.303277400067884	0	0
**S100A7**	S100A9	0.664460623730972	0	0
**S100A7**	S100A8	0.642083229265513	0	0
**S100A7**	KRT6A	0.498242847254381	0	0
**S100A7**	S100A12	0.491813632816533	0	0
**S100A7**	SERPINB3	0.489903857688437	0	0
**S100A7**	PCSK1	0.428948719798641	0	0
**S100A7**	SPRR1B	0.426116479956263	0	0
**S100A7**	S100A7A	0.406426816918189	0	0
**S100A7**	S100P	0.395168492583142	0	0
**S100A7**	FABP5	0.380259880838759	0	0
**S100A7**	SERPINB4	0.379287231882078	0	0
**S100A7**	AKR1C2	0.374432796549161	0	0

**S100A7**	RHCG	0.373816998133778	0	0
**S100A7**	KRT17	0.358301417615967	0	0
**S100A7**	S100A6	0.347677276904852	0	0
**S100A7**	SLAMF9	0.345701166737399	0	0
**S100A7**	SPRR3	0.345441304407219	0	0
**S100A7**	RAET1G	0.343615838705122	0	0
**S100A7**	LY6D	0.325514140241702	0	0
**S100A7**	CSTB	0.308367041453722	0	0
**S100A2**	S100A7	0.306381863008609	0	0
**S100A7**	TREM1	0.305036365076322	0	0
**HLA-B**	B2M	0.670816839088308	0	0
**HLA-A**	B2M	0.645217017888766	0	0
**HLA-DRA**	B2M	0.547515080314672	0	0
**PSMB9**	B2M	0.539486087206122	0	0
**HLA-DPB1**	B2M	0.535760303684876	0	0
**HLA-DPA1**	B2M	0.526817825879605	0	0
**CD74**	B2M	0.516645581546743	0	0
**S100A4**	B2M	0.504010926147824	0	0
**HLA-E**	B2M	0.50223624322514	0	0
**RARRES1**	B2M	0.491380191577878	0	0
**HLA-DMA**	B2M	0.472653064438561	0	0
**C6orf15**	B2M	0.471317899520969	0	0
**HLA-DQB1**	B2M	0.469348168648262	0	0
**CYBA**	B2M	0.466078671510317	0	0
**TAP1**	B2M	0.463874694786876	0	0
**HLA-DQA2**	B2M	0.46200886194395	0	0
**LEMD1**	B2M	0.460061272587022	0	0
**SAA1**	B2M	0.458552467756178	0	0

**IFI27**	B2M	0.455006993946915	0	0
**HLA-DRB5**	B2M	0.454124296450365	0	0
**MSLN**	B2M	0.450512100613418	0	0
**ARPC1B**	B2M	0.449997543327606	0	0
**HLA-F**	B2M	0.441181947766662	0	0
**PRSS21**	B2M	0.415967112636335	0	0
**AC005152.3**	B2M	0.413527828529371	0	0
**C2orf40**	B2M	0.407751528007019	0	0
**HLA-DQA1**	B2M	0.40713953665047	0	0
**KLK6**	B2M	0.398240248682478	0	0
**HLA-DMB**	B2M	0.390717778758902	0	0
**C1S**	B2M	0.389349033524566	0	0
**NDUFC2**	B2M	0.384463605532134	0	0
**SDHD**	B2M	0.383843952288796	0	0
**FSCN1**	B2M	0.382113459808393	0	0
**PTRF**	B2M	0.380001865419822	0	0
**CAV1**	B2M	0.379390157384983	0	0
**CTSC**	B2M	0.377756508004229	0	0
**MIA**	B2M	0.37749155000082	0	0
**RP11-54H7.4**	B2M	0.377403060333473	0	0
**HLA-DRB1**	B2M	0.377374343741693	0	0
**PTPRZ1**	B2M	0.376421260883323	0	0
**PLCXD3**	B2M	0.374933022933388	0	0
**FAM3C**	B2M	0.370306432252809	0	0
**COL8A1**	B2M	0.366955194787504	0	0
**CALB2**	B2M	0.365291788341178	0	0
**SAA2**	B2M	0.36377015847792	0	0

**AHNAK2**	B2M	0.363633516052269	0	0
**TMEM123**	B2M	0.363511484885992	0	0
**BST2**	B2M	0.361809961643752	0	0
**MFGE8**	B2M	0.361174998555508	0	0
**TMEM213**	B2M	0.360405422088346	0	0

Supplementary Table 3. The drug effects on selected therapeutic peptides

	BST2	GAPDH	S100A8	S100A9	H2AFJ	SCGB2A1	HMGN2	B2M	HLA-B	HLA-C
mitoxantrone	-3.06521416491631	0	1.30475361164477	0.440336824354434	-0.0379816476236297	0.286847933402364	-0.00344587920542905	0.0717156627112669	0.584912886052932	0.515357395297122
BRD-K34974324	-2.81575302237372	0	0.740002784209381	0.552715953621301	-0.222219644223354	0.20211933421511	0.117433883153939	0.0989991413608928	0.585278056182026	0.447425855335146
SKF-86002	-2.79909559680004	0.000194175746770053	-0.243946200031246	-0.709695178302811	0.430175110606677	0.348245141920095	-0.121482237630655	0.0872333798067886	-0.559792035738433	-0.433421958076007
oxalomalic-acid	-2.79022272310527	0	0.596333450732236	0.0896444005928272	0.119262253644663	0.0634810616834751	-0.0362150291806853	-0.00965870010312475	0.404286734955416	0.396741294006563
BRD-K05593511	-2.79022272310527	0	0.526810569174835	0.183276593561038	-0.0873329919659285	-0.0871866352559163	0.0844719043219633	-0.0393332417427428	0.420229858238089	0.340476274532263
ivermectin	-2.74137192669028	-0.0602417384276834	0.383336500922326	-0.380006283504612	-0.234116476838655	0.135843623618036	0.0704592099729626	0.0223612793182086	0.445419940849879	0.447836581874194
bezafibrate	-2.68881691423806	-0.0203905640004436	1.05855091827593	0.0582155194100005	-0.1343102864888	0.154500450484858	0.0469864083802722	0.0202469164026597	0.411148771100783	0.305978229183442
flumethasone	-2.66248676738365	-0.0437388906612481	0.243318958159139	-0.3963309060743	-0.0854782665858922	0.0321753127437265	0.0227322700631385	-0.034371528330559	0.221921343040081	0.140375703866492
BRD-K67306351	-2.64829692807301	-8.49794125024061e-06	0.347108074139008	-0.080041219219166	0.0847388742552093	0.406272238508917	0.0100845543408741	-0.0380370111130071	0.217775141824378	0.232994848423322
BRD-K21165668	-2.64068526184703	0	0.582774548144365	0.199227692137191	0.0536056513290366	0.121812917257606	-0.0107301962447339	-0.0261944044344968	0.348089953225322	0.200532404183948
AZD-5438	-2.62988402051617	1.63379340651915e-05	0.306093961814076	0.139053646435735	-0.00130931366299825	0.422991209335589	0.0726490482936653	0.0843069649825408	0.465793446638113	0.496264684159217
BRD-A97035593	-2.58831920609455	-0.0360387481497222	0.225488333898315	-0.128813818947549	-0.0605420022380749	0.240913901139312	0.041758713425827	0.0249050295623894	0.244478346945625	0.112752453308036
medroxyprogesterone	-2.56774995620905	2.09023911157402e-06	0.535038710120958	0.32743461253818	-0.100584314538146	0.200492520236108	0.109843895442814	0.0704575346314416	0.314675400481423	0.102007203665919
tolazamide	-2.56774995620905	2.09023911157402e-06	0.472983954093497	0.148220779149579	-0.111751984585992	0.14577488986676	0.114211572366794	0.140699251345826	0.446155102177226	0.363195750547217
BRD-K41303952	-2.56670646813885	-8.57330967196646e-06	0.637059914907793	0.448695987047914	0.0151807233406052	0.308142333040351	0.0350822485990117	-0.0966516198518046	-0.00462816143458733	0.0867066276332498
bucladesine	-2.54908047972945	-0.0564825725126625	-0.238681335488385	-0.429626297518509	0.339635638966946	-0.410113239848507	-0.0296071676080802	0.161790437670873	-0.0887269164095938	-0.570119798126168
abiraterone	-2.53423885498196	-2.4672798115688e-06	0.544879875060459	0.092808666193378	-0.0346796780618717	0.411747306947409	0.128842086044325	0.083221521513654	0.219490915418624	0.220746609024921
5-iodotubercidin	-2.53061880295225	0	0.596333450732236	0.444938539188102	0.124800771984075	0.235244284029132	-0.00511689227809464	0.170427828024704	0.575521351801112	0.351874405211047

BRD-A68890828	-2.50032789007931	0.046754492314097	0.344255203606002	0.0473475391574594	0.0780911087073681	-0.238334897790382	0.0212351462372213	0.0412280930732942	0.397383860712704	0.28523802315461
BRD-K04156788	-2.50032789007931	-0.220735658159896	-0.276844862118694	-0.409387051886307	0.243297594096564	0.191573867105948	0.138228751371483	0.0438084084255115	0.186374149889795	0.115656816674733
mepivacaine	-1.60896992567448	-1.33876820193331	0.11984915980677	0.235696918562898	-0.0686306083027208	0.0592174031287152	0.0602527590732653	-0.0854837451220831	0.120913874512343	-0.0135833497876621
BRD-K30381304	-0.468454212023807	-1.1992798906304	0.268610368065696	-0.0551233808768949	0.00581118432646965	0.0982407750898329	0.026661816323061	0.0159516464935141	0.258393021064754	0.214485879158122
verteporfin	0.241388454182181	-1.18783113594666	-0.457378530815059	-0.0958377355547575	0.0632978027439735	-0.10242045859182	0.0339502059788508	-0.0196502252589181	0.0324474989988106	0.0457013466693268
mammea-a	0.00158611087234295	-1.17354469659949	-0.0651002653856523	0.029908864062778	-0.148160414059693	-0.425186125469352	-0.0601280764792436	-0.0440331079796277	0.0571521932361154	0.140172061145183
AM-281	0.109577851936891	-1.17278512513892	-0.243437173475193	-0.116470259672656	-0.0431057707196505	0.0399292928375314	-0.0576466313949751	-0.0677928624805948	-0.0998047542543494	0.0628890236327542
megestrol	-0.10133928768943	-1.17246661147934	0.128414429899527	0.123084787814325	0.00956607113396668	0.0624988262442008	-0.0106197887393376	-0.0717466150988106	-0.114936783210375	-0.0716106324530954
SYK-inhibitor	0.1832013677371	-1.16818049095711	0.124025238446937	-0.0193845640211334	-0.0185802429772286	-0.454465335002512	-0.0759459609432174	-0.0344702139102266	-0.0230233502739678	0.0209680851373801
mifepristone	0.143946802496091	-1.16220120695943	-0.168231039063966	-0.331144524247943	0.0473205953108584	0.0530668904706446	-0.117055445133821	-0.0569173409945298	0.066128820092652	-0.0478958543090013
EO-1428	-0.0223279544500197	-1.14819384827637	0.333736952944523	0.149274274421146	0.0162264630080324	-0.147158429456444	0.0717806351158816	-0.0953345388090874	0.155250775389493	0.0633184039641561
tamoxifen	0.103008840318316	-1.14138642361449	0.681274784561524	0.571914270146885	0.222758386652986	0.226473707273923	-0.263194979313021	0.0319098226912717	-0.0578108636128691	-0.0657658353259594
thapsigargin	0.0560119251742863	-1.13867728974296	-0.0926393530117706	0.143255762991856	0.0489742571090521	-0.155635370291278	-0.11623631615952	0.0159926029557971	0.0583809864171054	-0.0523666987282954
beclomethasone-dipropionate	0.198846881350625	-1.138241247025	-0.358676890202882	-0.518516200674381	0.0324169215089194	-0.63232679504776	-0.0222381864955894	-0.0196200380767206	-0.0396642185377312	0.00928521049219944
CP-93129	-0.0596204932983624	-1.13362934213998	-0.228841583672821	-0.181405824717392	0.135318881860853	-0.172339716863227	-0.00222846963319467	-0.0154548781081507	-0.180921766032476	-0.121551689279786
siguazodan	-0.15747233348139	-1.13309946388528	0.115815836800784	0.115664016701967	-0.0214566341811424	-0.226466270481771	-0.00775522103988946	0.0337001591319255	-0.0314691292614837	-0.0901065305309712
BRL-54443	-0.0250141732245912	-1.13236394294264	0.222909286345959	0.186801851657186	-0.0229945843878023	-0.147928731721426	0.0140587375279555	-0.133893316054015	-0.108991711563096	0.0201856066186936
YS-035	0.330874782560511	-1.1273584611264	0.192905358633504	-0.054511288190465	-0.0332508020291553	-0.337226915638379	-0.0486089430146017	0.001435184622542	0.0432534646403289	0.0758289110931667
lamotrigine	-0.0059621263414722	-1.12663237374364	0.303892020753401	0.0693203216193874	0.0107763968181565	0.138299545441439	-0.0101606046261775	0.0130110280323135	0.0814538922878958	0.0833894165120976
tetramethylsilane	-0.133120768079624	-1.12627167858252	-0.331231446297561	-0.164654665537373	-0.0277221431995041	0.0159410538970945	0.0105692628179268	-0.0420875322627903	-0.075077064299141	-0.0908441637792654
homatropine	0.0968172001391467	-1.12574773634846	0.222059581011354	0.084138879963874	0.0605711059046845	0.170112278637875	-0.0514365239137828	-4.66852457758904e-05	-0.00682775815460257	0.0260785875914955
piretanide	-0.0475388503888436	-1.12551512211624	0.130190551114966	-0.032546990872119	-0.0449554488844952	-0.508724008136618	0.0913214448100734	-0.131862386912665	0.127808295749816	0.146378916841336
melperone	0.565233237180566	-0.10236862115413	-3.54463499595589	-3.2330382837125	-0.00905873754634401	0.228719405422055	-0.0360534391042826	0.157304436362923	-0.811805093588194	-1.17276266684379
cefpodoxime	0.177789392780664	-0.0167462332357329	-3.36614343551925	-1.65850076629079	-0.199920573417246	0.322153391474815	0.109369048886766	0.153181464083847	-1.24222409714729	-0.812938966340119
stavudine	1.08531287824623	0.00916979826110964	-3.21528382199103	-1.06671438127351	0.0262835691172985	-0.0503785613791154	0.0465120190400041	0.215714377920845	-0.231245728589213	-0.382318606385017
kitasamycin	-0.313942158207076	0.00144672660698886	-3.07110781359692	-1.93937348785373	0.00966932217882377	-0.0330626293592915	0.097839732730121	-0.115935108163085	-0.0793829600106837	0.223755435490512
purvalanol-a	0.394798235647968	-0.0374086907848503	-3.04189295088266	-0.853846180168267	0.0930798074899442	0.000400494786564121	-0.00289518163756375	0.0619856220434949	-0.183788274981678	-0.163387488556946
alprazolam	1.0174642025265	0.00144672660698886	-2.74091200147747	-1.51595965074301	-0.239821430257821	0.241705668074652	-0.0403263192776948	0.0938086364659081	0.217501224338476	-0.0135432578100438
pergolide	-0.0908187507169105	-0.106978507655452	-2.56578041815509	-1.66393609091301	0.0900866663860818	0.250896769625267	-0.0812409825695593	-0.0895730692751671	-0.37895196146245	-0.635235281401398
estriol	0.388524293169398	-0.287798423079373	-2.43342687683289	-1.04473116973005	0.157297018458043	0.095041770000722	-0.0577347352558482	-0.0144636399591476	-0.3542659494262	-0.271233347318885
BRD-A69421747	-0.00948457185996787	3.28328833987257e-06	-2.3300961833849	-0.965102861684666	-0.0203413227631839	0.0560785353667983	0.0551062263248321	-0.0077577711786132	-0.174223764728738	-0.0971710534011434
PRISM001_MCF7_ 24H_X1_ B7_DUO52HI53LO:G03	0.314371835101897	-0.0241717780392525	-2.26610973393463	-1.7940381879705	-0.0170785280904093	0.0938131899430705	-0.0432535427052296	-0.274943160245126	-0.378472974152741	-0.184202634984111
MDL-73005EF	-0.214138278763997	-0.101845505998329	-2.25763561634976	-2.61526963521825	0.0806738814150738	-0.433954599265714	-0.111953434456731	0.118759890957135	-0.280281251081706	-0.61775944849038

ZSTK-474	-0.408839338635179	0.0400859139525074	-2.20065900199572	-1.03873809219199	-0.0312474978270401	0.0138306129152879	-0.0221478837080653	-0.227885033368383	-0.245478391477348	0.0636875607255809
cyclopentene	0.0993302598627079	-0.0578434407409198	-2.14188604157172	-1.25214469061655	0.187947885133056	-0.183120276358023	-0.0789317331320158	-0.00987310857077617	-0.13064638653855	0.0660085437758959
SU-11652	0.45005033413438	0.0174338755388215	-2.14003542082683	-1.34138052063423	-0.0224547496970033	0.00961948858246009	-0.076831868015792	0.00408077889254654	0.137922068280086	0.0806600834418076
azlocillin	0.899169797112558	-0.0180962023832931	-2.13336484703589	-0.336131035522768	0.0445481339563996	-0.128345024179716	-0.0147060444075477	0.17010715569524	-0.361488903638036	-0.463925539203158
cefepime	0.755297070547492	0.00193140847581752	-2.13336484703589	-0.91672919950917	0.00557990776538997	-0.0346450307089401	-0.00842232346826277	0.182072870595761	-0.22033572738468	-0.365889670228005
tamibarotene	0.0776986254430172	0.0471317250783425	-2.12423629159841	-0.873445625391351	-0.0693548484907973	-0.398269822116042	0.0507297125852348	-0.295471891602111	-0.21030079215581	-0.112838825498356
altanserin	-0.0161545807011305	-0.125192806276249	-2.12148714774397	-1.11344969193042	0.0178437348650275	-0.940579792342502	-0.0989810705469938	-0.199218645580534	-0.736246930843865	-0.427620759652281
wortmannin	0.857318045901151	-0.010608325426674	-2.06683780728741	-0.427728553726657	0.0740562252020949	-0.0843213660351956	-0.0896392436039175	0.145712584857964	-0.352737906842279	-0.443684019970344
fenipentol	0.587967076059915	0	-1.9985216991278	-0.731501046930581	0.161450850965345	0.00547693118437875	-0.022463092969951	0.012022943028839	-0.0357290584840544	-0.00768927394093621
nicorandil	0.521631193028896	-0.05264601646441	-1.06522327856298	-3.2330382837125	-0.209420272307688	0.340346351317537	-0.0138131001559012	0.203503135573617	-0.130873188809698	-0.0940212258863422
BRD-K20168484	1.22123528332008	0.00144672660698886	-1.63413261158508	-3.2330382837125	-0.0104227860295087	0.340346351317537	-0.22896202713482	0.0681347540324677	0.368649374694806	0.0921074397691046
halometasone	-0.489707697036994	-0.0880028550852812	-1.41607759410623	-3.2330382837125	0.0796341343656919	-0.0817294227946372	-0.0212190728833757	0.118759890957135	-0.373633615359353	-0.561492329156879
tacrolimus	0.47718018083	-0.00756210456219275	-0.929571851482954	-3.2330382837125	0.227967280360806	0.331337520148355	-0.0421003609929147	-0.00658327052604646	-0.314690575293067	-0.0866712093894964
SC-19220	-0.346167927238714	0	-0.84997076288992	-2.60919189122827	0.0364383527525911	0.0388553348453775	-0.0208916456765218	0.157453565490364	0.264168328353672	-0.801562179527717
raloxifene	-0.0976850528728979	-0.535956973246901	-1.3279499188552	-2.4704586904941	-0.200457572479796	0.146821525482707	-0.0398832621880163	-0.328628398165018	-0.272630283616255	-0.0105612866652112
toremifene	0.329004554458606	0.203017957361857	0.210388187901328	-2.30020062495464	-0.166164349479594	0.0197688502982598	-0.0219119770323299	0.0271085017748476	0.0542030301715632	0.10755810669804
indatraline	-0.54319888268141	-0.0747765138516225	-1.76155553074228	-2.23145218599852	0.346304295920067	0.263096057722498	0.051959310020143	-0.355265200683135	-0.520386838415894	-0.351882104701095
wortmannin	0.310107985401461	-0.0435729860396736	-1.35352589538209	-2.14992394088091	-0.0848442611398648	0.0357350148120208	-0.032258635582517	-0.0724836808621574	0.0194376235953873	-0.00182454105327601
taxifolin	0.347586000870701	0.00144672660698886	-0.377004155268213	-2.14890595433938	0.353634203191272	0.241019996178332	-0.152271042402081	0.266344923668409	-0.130046985433377	-0.389886057264818
BRD-K71746704	0.292425118521411	-0.116435703748046	-1.04166499292205	-2.12942365755624	-0.340959845034941	0.296570816325852	-0.0459165957607302	0.389229879566147	0.68595859406811	0.113504199394815
BRD-K69304050	-0.389096206338515	0.0391830340616108	-1.0379173085056	-2.09972127417229	0.432943916443912	-0.146474492925988	0.0489249252222699	0.231474473011457	0.161760733297843	-0.506949728959125
sorafenib	-0.511960653968247	0	-1.05217007680634	-2.09120786524577	0.0287137073802857	0.423464767558964	-0.0863383724088478	0.36706889085814	0.798995887600582	0.225325396066816
staurosporine	-1.51099153578432	-0.0697029161643568	-0.706463968491749	-1.9776549682399	-0.0546430665609128	0.249341856511841	0.107449195307767	-0.00916867956909995	0.133693804550022	0.076912483392872
ethinylestradiol	0.438793152535774	-0.00647370856021512	-0.873568133496531	-1.92444441623914	-0.135222440804979	0.10205714947601	-0.00406824775054249	0.294991781775642	-0.135248181632075	-0.168421646213314
BRD-K21001652	0.126678913260009	-0.125020495818757	-1.05545961588331	-1.89276607065339	-0.0874166350470964	-0.0154733994874405	-0.0504112914085817	0.0678621423036412	0.066811757443987	-0.0820808477926596
vincristine	0.402088321849546	-0.047492449262617	-0.613434180397876	-1.87211218645828	-0.119861990158104	-1.40832843114995	-0.00434583038864389	0.196190286957362	0.321194641409039	0.270932234136383
BRD-K15206810	-0.502718649607072	-3.97466724688655e-06	0.56520224897925	0.568939562022615	0.642075659158332	0.184130573239784	-0.285782224460425	0.162621888124126	0.40045636966307	-0.187417307876601
bicalutamide	0.327156659567292	-0.10550176295116	-0.206196331591689	-0.546717083798705	0.633894813711771	-0.403488304371663	-0.0167709697205982	0.304492285873292	-0.358417089802986	-0.557172840549586
trifluoperazine	0.833065485689517	-0.0167462332357329	0.0266899209244409	-0.589759300614078	0.575248888533167	-0.470034616641464	-0.199272412153154	0.128347910541062	-0.380775932269648	-0.219396756643422
artemether	-0.316167685169968	-0.111671528603798	-0.925785542484106	-1.11344969193042	0.574371052231133	-0.996331825138506	-0.166904214316429	-0.100100677883586	-0.752080086351745	-0.838057260296043
BRD-K59036917	-0.620429519359678	-0.0635059499719119	-1.03689392183571	-1.79633629030437	0.559280830087641	0.258611444717003	-0.0150165516566738	0.176459141872319	-0.195460253115716	-0.61526908999121
BRD-K78716413	-0.63336253048188	-0.0373846811701628	0.40068608351511	-0.79058889300241	0.540590108176445	0.227694165312886	0.0535704558248651	0.212188263059833	0.185675522319134	-0.3315335109942
BRD-K53638321	-1.49159806592597	-0.0876003567635828	0.691862178082679	-0.321517163582838	0.540231795160786	0.286917199995453	-0.214990043727199	0.185849466744822	-0.230915067512754	-0.382052728353955
BAY-11-7082	-1.18319044305023	-0.0388750571081014	-0.390336867971538	-1.28427158945943	0.523353960293092	0.260019782676948	-0.0542632815109951	0.0310006137636787	-0.199826761146851	-0.515771635399123

FL-HDAC-025	0.119269874580362	0.0280900419254166	1.73331139749178	1.22597453495349	0.517121398738495	-0.576881672979602	-0.012790912213458	0.17185778716059	0.296748349374151	-0.0203574150634078
BRD-K03552198	-0.279372232638427	2.12110764419293e-06	0.331492309933688	0.0493666830868618	0.515934855419852	-0.279582307780742	0.153579232203465	0.0700643215324712	0.202093795334635	0.355089238906029
pirenperone	0.463880163622167	-0.0782123222792199	-0.745392214082564	-0.466019140024188	0.491840074275979	-0.154029481438549	-0.155942130744307	0.188449094479651	-1.42629507915616	-1.05337119325836
trazodone	0.02184794162548	-0.0722406269694846	0.041793427797006	-1.51232203952164	0.490289487427095	0.340346351317537	-0.0382156022842097	0.0515000669819994	-0.249533397619196	-0.216817116523053
eucatropine	-1.93982439682347	0.00356130616072203	0.214125515176088	0.0378819931849841	0.489095544987142	0.256118440004721	-0.350421212314314	0.261563260484635	0.575616007287314	0.556907522227442
PRISM001_MCF7_ 24H_X1_B7_ DUO52HI53LO:M05	-1.39900141517768	0.0482862279792786	0.748463901955916	-0.176971321939078	0.485279447487355	0.332314731953545	0.0501285949627261	0.11319246649481	-0.147945019723069	-0.462675911735032
BRD-K77432048	-0.689915117504993	-0.0617861262185206	0.189301861751111	-0.362300586978505	0.470634970398673	0.437850614187409	-0.0717104069460088	0.089194291125835	-0.371929571964474	-0.574354788569583
PCLB003_MCF7_ 24H_X1_B13:D07	-1.86858966595719	0.153801066700452	-0.497460490920921	-0.945413892269384	0.456194393123708	0.143899885647983	-0.106302289351119	0.115892145852864	0.424379607765144	-0.0139923782803124
tegafur	0.655449123533416	-0.170845988535481	-1.95429648617083	-0.830043298968345	0.445348901567676	0.245042339253116	-0.0842389541209871	0.165117737982054	-1.13848865707049	-0.839505881598825
bromocriptine	-0.558630647466166	0	0.0592009662947941	-0.151103230709428	0.441572377231932	-0.164548865894673	-0.117371595958788	0.145720960105789	0.180766494428685	0.15610031707473
BAY-11-7082	-0.26862043060573	-0.0442728216198782	0.510811751329184	-0.783571986986491	0.435760185562315	0.231658936954964	0.0894574674168669	0.212487518415165	0.0454226986926924	-0.470521035122528
BRD-K92158425	-1.15027668891999	0	0.597974397271727	0.336354240699015	0.0317872634869452	0.724183439918484	-0.0282437614424538	0.189858549143179	0.379563474212874	0.387466819910341
BRD-K53780220	-0.80675077143724	0	0.772890909296959	0.543649820127	-0.131056452150275	0.672676597022718	-0.00981833327870252	0.156228816134815	0.468443332872898	0.483046726840769
CAM-9-027-3	-0.531832318436316	0	0.320939041969461	0.0125270121000698	-0.0578106024929026	0.661168972606341	0.0230587194879779	0.0317502956722966	0.0408488893728105	0.181497772058412
CS-110266	-0.926197294020916	0.00969617649269328	0.601901928811072	0.318265067603229	0.0370895089147365	0.627735565833865	0.00905666130673666	-0.0380679874743106	-0.11877772570667	0.0746150212125327
VU-0418946-1	-0.447334334519369	0	0.472697910065342	0.282468067233703	-0.0244859292743973	0.621941797952034	-0.0990182484164275	0.0440498052259954	0.339407026030001	0.28640989366908
VU-0415113-1	-0.513372747204959	-0.010018517329407	0.510718257482092	0.0833764400305963	-0.0659283399305868	0.615936301866525	-0.040371017339297	-0.0333174084767802	-0.183791096147883	-0.033464163786165
BRD-K25536815	-0.5239109087973	-4.47149988236362e-06	0.361494614038253	0.28413828781376	-0.0670530085586951	0.611324426381034	0.0107952832510763	-0.0105797796386162	0.00508172504863369	0.0676314594221403
BRD-K01896723	-0.505291935985575	0	0.629055003575443	0.441083566830312	-0.0294297035828652	0.577375892539678	0.0313181409226715	0.113272604294911	0.395030165414885	0.426556307018299
I-505056	-0.0767049918771683	2.09023911157402e-06	-0.100141334822453	0.334331158184798	-0.212922946714687	0.575102523866686	-0.0518329691349892	0.234378058839559	0.65906402309939	0.186464561796111
doxazosin	-0.163923295157691	-6.93882180424765e-06	0.47530366630191	0.357558089800069	0.208446646648131	0.56418237469791	0.0241176640467566	0.230604307031085	0.0963582071583322	-0.00408369589914415
KU-C103885	-0.343547898269234	-4.47149988236362e-06	0.31353819629182	0.0444858230781784	-0.176852662945461	0.562121450625328	0.01344934711289	0.00805234436857782	-0.00185501042837588	0.0454142053079978
BRD-K08339178	-1.13968797482513	2.09023911157402e-06	-0.824386026391413	-1.27954298454755	0.150574354189921	0.55997568707004	-0.0285033296407695	0.305713215298348	-0.496857750424528	-0.755615513462747
BRD-K95337198	-0.67715151522807	-4.47149988236362e-06	0.510236878938293	0.232177673410708	-0.280760442688777	0.558779770258113	0.0338450933090457	0.0916697962570727	0.213017389682935	0.0831003359123099
BRD-K77681376	-0.39265689474641	0	0.74487581147449	0.502417681725309	-0.080831025563123	0.556509619033613	-0.0904088647408501	0.155537699696093	0.451939712130137	0.389859700558421
NCH-51	-1.90285758183134	2.09023911157402e-06	0.322774830789521	-0.176434505497106	0.00823425177867065	0.554622771160709	0.0549196460120136	0.166049639602331	0.509762773139633	0.344028173667896
cyclophosphamide	0.201974107249656	-6.93882180424765e-06	0.202539829482431	0.459979780793413	-0.0891573125361651	0.551493160350864	-0.0707086390388478	0.185001121335969	-0.000449578302483378	0.166593689850038
CAM-9-021	-0.36439206145179	0	0.501105300939171	0.164138387605541	0.00113217759384598	0.547868154003969	0.0236205141704819	-0.0164162859181456	0.252024276534418	0.237873407347966
naltrindole	-0.663283952342637	-0.114028554366267	0.694290506234168	0.301510672543934	-0.287388522996853	0.541429129966721	0.0371490509048931	-0.0990929730156678	0.28163606867291	0.139306837252256
BRD-K39318886	-0.597512010301028	0	0.227803759483892	0.162671937569862	-0.0515657147712285	0.53961552451679	-0.0104755732774646	0.020807636819431	0.213751324823733	0.0930802685284293
BRD-K74777906	-0.18104340867248	0	0.235532216159275	0.0096952801239536	-0.137893345540767	0.535147751768062	-0.0151868457400428	0.0904447827162351	0.164632607162636	-0.0244772231651278
ketotifen	0.570850707048847	0.423449241259754	0.10095784252246	0.524489379950379	0.168475988757354	-0.139141123956271	0.234426395538389	0.181964692444653	0.126604653493355	-0.049930747240579
wortmannin	-0.244668919416757	0.296151589225904	0.0046484497869046	0.00800085901594949	-0.42547902298731	-0.0700123633874172	0.229894197517767	0.209934788174725	-0.0694415676297422	-0.274821718641843

benfluorex	-0.878252644966231	0.10870440892317	-0.0747002537893122	0.262850287321644	0.135469658389996	-0.0552848091957765	0.21641196013317	0.168650666088877	-0.661973504291896	-0.607218244697048
parthenolide	-0.955221125603092	0.161131192179253	0.158447376476433	0.399913223300918	-0.0442540995353773	-0.166450637666205	0.199105309452245	0.324112488752082	0.0815435308838559	-0.263233664836799
SCH-23390	-0.183959194072335	0.139209290350025	-0.320653673478668	-0.138235289286409	-0.0423719141817873	0.0126991527299429	0.196799287649468	0.0106938325849302	-0.527251190197908	-0.279819472639809
mebendazole	-0.081459869378303	-0.249797934751213	0.176904899330393	0.356915717279283	0.0282775700061095	0.0152711344795193	0.195726009912431	0.0663926168092894	0.131804113222655	0.00751029728269392
BRD-K45232279	-0.852612926431465	0.0177869289977095	-0.0854132568112522	-0.276292321250174	-0.155105298693554	-0.0345355044383271	0.188705385143245	-0.0226728230595112	0.210734093226191	0.113018157955848
hemicholinium-3	0.812471130419732	0.360643341741129	-0.124444396440076	0.113004117481383	-0.288657244859256	-0.0706426734674257	0.187814394110726	0.246567821191566	-0.313333598478597	-0.364637765199961
BRD-K32112425	-1.27153483209068	0.0375667157555863	0.502819556595904	0.192889232847083	-0.0228957967339367	-0.278509255296835	0.183607723740253	0.136478146620709	0.216891139477598	0.0437547170285022
kinetin-riboside	0.218594783119287	0.469589052692549	-0.0879431288854269	0.0507970342465072	-0.128335190212074	0.0126975592145948	0.182087596207949	-0.0546407202997252	-0.279289681732911	-0.24629566430663
SA-247714	-0.969647836918387	0.0928052396268111	-0.0676302527643484	-0.244881967933679	-0.049686874508367	-0.0164616398719541	0.181243802157743	-0.157733672901658	-0.0110573251184478	-0.0370671187756879
nitrendipine	-0.25247210451921	-0.0243822458407092	-0.86458951482827	-0.55006635968926	0.0806587666785341	0.190150327062876	0.180697902304416	0.031622047124936	-0.0772068342387437	-0.190554704553062
BRD-K32927008	-1.30902711874284	0	0.471361497788208	0.150970929872166	-0.130586900561586	0.0803589828657576	0.178021878612848	0.0122422036157044	0.504956430341855	0.203938461941222
BRD-K63979920	-0.499453501141135	-0.0233472154742822	0.439200724001624	0.294663561837996	-0.230865477058139	0.0260122583683069	0.16676679316351	-0.0733663090120595	0.488135179192606	0.438025117631471
mepacrine	-0.500806142648131	0.0111407293329666	0.474279042194055	0.192369312130609	0.0311977197526794	0.0561981183111131	0.163934567990776	-0.0765192585275236	0.169509532189279	0.204563815160348
sulfachlorpyridazine	-0.0995362522489294	0.00218824612463031	-0.51266195098914	-0.125023653981275	0.0660533046563887	-0.0572274148815564	0.160923916582862	-0.0592224149795211	-0.0612011467556277	-0.0166068492455387
trichostatin-a	0.261225310319259	0.700241664804693	0.227917598072284	0.544392209689759	-0.386020423183711	0.288854319035771	0.158963068538613	0.217977176901643	0.434081097724338	0.528011726744365
BRD-K17025677	-0.927929160106363	-0.0106336969722869	-0.356061522449559	-0.364667163660837	-0.0625079063174714	0.0126991527299429	0.158797489318003	-0.0944054087030604	-0.287045533558886	-0.070452723345992
tropisetron	1.42373334668744	0	0.372319162580656	0.62484808590514	-0.00935774638701936	-0.101618402637107	0.158687524457109	0.0435696958426468	-0.247387496040191	-0.131091229902324
EMF-sumo1-6	-0.283341581030756	0	0.0578522412577174	-0.299165780523086	-0.07841034369683	0.230979765493778	0.1583097490176	0.0371320618806359	0.123607804206798	0.152632190886372

Supplementary Table 4. The metadata of the selected drugs

inst_id	rna_plate	rna_well	pert_id	pert_iname	pert_type
CPD001_MCF7_24H_X1_B6_DUO52HI53LO:N01	CPD001_MCF7_24H_X1	N01	BRD-K21680192	mitoxantrone	trt_cp
CPC019_MCF7_24H_X4_B5_DUO52HI53LO:C23	CPC019_MCF7_24H_X4	C23	BRD-K34974324	BRD-K34974324	trt_cp
CPC016_MCF7_24H_X4_B5_DUO52HI53LO:C20	CPC016_MCF7_24H_X4	C20	BRD-K96809896	SKF-86002	trt_cp
CPC014_MCF7_24H_X1_B5_DUO52HI53LO:P23	CPC014_MCF7_24H_X1	P23	BRD-A80574334	oxalomalic-acid	trt_cp
CPC014_MCF7_24H_X1_B5_DUO52HI53LO:P19	CPC014_MCF7_24H_X1	P19	BRD-K05593511	BRD-K05593511	trt_cp
CPD001_MCF7_24H_X3_B6_DUO52HI53LO:O14	CPD001_MCF7_24H_X3	O14	BRD-K24652731	ivermectin	trt_cp

CPD001_MCF7_24H_X3_B6_DUO52HI53LO:L24	CPD001_MCF7_24H_X3	L24	BRD-K46018455	bezafibrate	trt_cp
CPD002_MCF7_24H_X2_B6_DUO52HI53LO:C24	CPD002_MCF7_24H_X2	C24	BRD-K81774264	flumethasone	trt_cp
CPC007_MCF7_24H_X1_B5_DUO52HI53LO:J22	CPC007_MCF7_24H_X1	J22	BRD-K67306351	BRD-K67306351	trt_cp
CPC012_MCF7_24H_X1_B5_DUO52HI53LO:P11	CPC012_MCF7_24H_X1	P11	BRD-K21165668	BRD-K21165668	trt_cp
LJP002_MCF7_24H_X3_F2B5_DUO52HI53LO:K15	LJP002_MCF7_24H_X3	K15	BRD-K72414522	AZD-5438	trt_cp
CPC007_MCF7_24H_X4_B5_DUO52HI53LO:P20	CPC007_MCF7_24H_X4	P20	BRD-A97035593	BRD-A97035593	trt_cp
CPC020_MCF7_24H_X5_B5_DUO52HI53LO:P14	CPC020_MCF7_24H_X5	P14	BRD-K82216340	medroxyprogesterone	trt_cp
CPC020_MCF7_24H_X5_B5_DUO52HI53LO:O18	CPC020_MCF7_24H_X5	O18	BRD-K32164935	tolazamide	trt_cp
CPC013_MCF7_24H_X2_B5_DUO52HI53LO:P12	CPC013_MCF7_24H_X2	P12	BRD-K41303952	BRD-K41303952	trt_cp
CPC006_MCF7_24H_X4_B4_DUO52HI53LO:K23	CPC006_MCF7_24H_X4	K23	BRD-A04706586	bucladesine	trt_cp
CPC020_MCF7_24H_X5_B5_DUO52HI53LO:K18	CPC020_MCF7_24H_X5	K18	BRD-K55301415	abiraterone	trt_cp
CPC014_MCF7_24H_X1_B5_DUO52HI53LO:N19	CPC014_MCF7_24H_X1	N19	BRD-A18497530	5-iodotubercidin	trt_cp
CPC007_MCF7_24H_X2_B5_DUO52HI53LO:P22	CPC007_MCF7_24H_X2	P22	BRD-A68890828	BRD-A68890828	trt_cp
CPC007_MCF7_24H_X2_B5_DUO52HI53LO:M24	CPC007_MCF7_24H_X2	M24	BRD-K04156788	BRD-K04156788	trt_cp
CPD003_MCF7_24H_X3_B6_DUO52HI53LO:B24	CPD003_MCF7_24H_X3	B24	BRD-A03216249	mepivacaine	trt_cp
CPC012_MCF7_24H_X2_B5_DUO52HI53LO:K18	CPC012_MCF7_24H_X2	K18	BRD-K30381304	BRD-K30381304	trt_cp
CPC017_MCF7_24H_X1_B4_DUO52HI53LO:I20	CPC017_MCF7_24H_X1	I20	BRD-M45964048	verteporfin	trt_cp
CPC016_MCF7_24H_X3_B4_DUO52HI53LO:N23	CPC016_MCF7_24H_X3	N23	BRD-K92138166	mammea-a	trt_cp
CPC005_MCF7_24H_X2_B4_DUO52HI53LO:F01	CPC005_MCF7_24H_X2	F01	BRD-K59419204	AM-281	trt_cp
CPC005_MCF7_24H_X2_B4_DUO52HI53LO:L01	CPC005_MCF7_24H_X2	L01	BRD-K19507340	megestrol	trt_cp
CPC017_MCF7_24H_X1_B4_DUO52HI53LO:J21	CPC017_MCF7_24H_X1	J21	BRD-K30189597	SYK-inhibitor	trt_cp
CPC016_MCF7_24H_X3_B4_DUO52HI53LO:D11	CPC016_MCF7_24H_X3	D11	BRD-A44780397	mifepristone	trt_cp
CPC017_MCF7_24H_X1_B4_DUO52HI53LO:E21	CPC017_MCF7_24H_X1	E21	BRD-K42452249	EO-1428	trt_cp
CPC005_MCF7_24H_X2_B4_DUO52HI53LO:H10	CPC005_MCF7_24H_X2	H10	BRD-K04210847	tamoxifen	trt_cp
CPC005_MCF7_24H_X2_B4_DUO52HI53LO:L20	CPC005_MCF7_24H_X2	L20	BRD-K69023402	thapsigargin	trt_cp
CPC016_MCF7_24H_X3_B4_DUO52HI53LO:D13	CPC016_MCF7_24H_X3	D13	BRD-K97810537	beclomethasone-dipropionate	trt_cp
CPC016_MCF7_24H_X3_B4_DUO52HI53LO:L13	CPC016_MCF7_24H_X3	L13	BRD-K81876028	CP-93129	trt_cp
CPC017_MCF7_24H_X1_B4_DUO52HI53LO:K23	CPC017_MCF7_24H_X1	K23	BRD-A62071884	siguazodan	trt_cp

CPC016_MCF7_24H_X3_B4_DUO52HI53LO:E08	CPC016_MCF7_24H_X3	E08	BRD-K17868609	BRL-54443	trt_cp
CPC017_MCF7_24H_X1_B4_DUO52HI53LO:A23	CPC017_MCF7_24H_X1	A23	BRD-K06208435	YS-035	trt_cp
CPC016_MCF7_24H_X3_B4_DUO52HI53LO:D09	CPC016_MCF7_24H_X3	D09	BRD-K93460210	lamotrigine	trt_cp
CPC016_MCF7_24H_X3_B4_DUO52HI53LO:P19	CPC016_MCF7_24H_X3	P19	BRD-K45988865	tetramethylsilane	trt_cp
CPC005_MCF7_24H_X2_B4_DUO52HI53LO:A22	CPC005_MCF7_24H_X2	A22	BRD-A09539288	homatropine	trt_cp
CPC016_MCF7_24H_X3_B4_DUO52HI53LO:H15	CPC016_MCF7_24H_X3	H15	BRD-K87990216	piretanide	trt_cp
CPC011_MCF7_24H_X4_B5_DUO52HI53LO:D24	CPC011_MCF7_24H_X4	D24	BRD-K92984783	melperone	trt_cp
CPC011_MCF7_24H_X4_B5_DUO52HI53LO:M14	CPC011_MCF7_24H_X4	M14	BRD-A87387433	cefpodoxime	trt_cp
CPD003_MCF7_24H_X3.L2_B6_DUO52HI53LO:M22	CPD003_MCF7_24H_X3.L2	M22	BRD-K93880783	stavudine	trt_cp
CPC011_MCF7_24H_X4_B5_DUO52HI53LO:E22	CPC011_MCF7_24H_X4	E22	BRD-K96134740	kitasamycin	trt_cp
CPC005_MCF7_24H_X5_F2B4_DUO52HI53LO:B23	CPC005_MCF7_24H_X5	B23	BRD-K50836978	purvalanol-a	trt_cp
CPC011_MCF7_24H_X4_B5_DUO52HI53LO:A10	CPC011_MCF7_24H_X4	A10	BRD-K32398298	alprazolam	trt_cp
CPC011_MCF7_24H_X4_B5_DUO52HI53LO:H24	CPC011_MCF7_24H_X4	H24	BRD-K60770992	pergolide	trt_cp
PCLB003_MCF7_24H_X1_B13:D07	PCLB003_MCF7_24H_X1	D07	BRD-K17016787	estriol	trt_cp
CPC009_MCF7_24H_X5_B5_DUO52HI53LO:E16	CPC009_MCF7_24H_X5	E16	BRD-A69421747	BRD-A69421747	trt_cp
PCLB002_MCF7_24H_X3_B13:C07	PCLB002_MCF7_24H_X3	C07	BRD-K52160314	BRD-K52160314	trt_cp
CPC011_MCF7_24H_X4_B5_DUO52HI53LO:I24	CPC011_MCF7_24H_X4	I24	BRD-A50764878	MDL-73005EF	trt_cp
CPC014_MCF7_24H_X5_F2B4_DUO52HI53LO:C23	CPC014_MCF7_24H_X5	C23	BRD-K63068307	ZSTK-474	trt_cp
PCLB003_MCF7_24H_X3_B13:M19	PCLB003_MCF7_24H_X3	M19	BRD-K98109757	cyclopentene	trt_cp
CPC006_MCF7_24H_X3_B4_DUO52HI53LO:M15	CPC006_MCF7_24H_X3	M15	BRD-K60623809	SU-11652	trt_cp
CPD003_MCF7_24H_X3.L2_B6_DUO52HI53LO:E03	CPD003_MCF7_24H_X3.L2	E03	BRD-K73437736	azlocillin	trt_cp
CPD003_MCF7_24H_X3.L2_B6_DUO52HI53LO:P18	CPD003_MCF7_24H_X3.L2	P18	BRD-K58662656	cefepime	trt_cp
CPC014_MCF7_24H_X5_F2B4_DUO52HI53LO:P18	CPC014_MCF7_24H_X5	P18	BRD-K36627727	tamibarotene	trt_cp
CPC011_MCF7_24H_X4_B5_DUO52HI53LO:D20	CPC011_MCF7_24H_X4	D20	BRD-K00610438	altanserin	trt_cp
CPD003_MCF7_24H_X3.L2_B6_DUO52HI53LO:A04	CPD003_MCF7_24H_X3.L2	A04	BRD-A75409952	wortmannin	trt_cp
CPD002_MCF7_24H_X2_B6_DUO52HI53LO:L05	CPD002_MCF7_24H_X2	L05	BRD-A66284731	fenipentol	trt_cp

CPC011_MCF7_24H_X4_B5_DUO52HI53LO:N16	CPC011_MCF7_24H_X4	N16	BRD-K97752965	nicorandil	trt_cp
CPC011_MCF7_24H_X4_B5_DUO52HI53LO:N18	CPC011_MCF7_24H_X4	N18	BRD-K20168484	BRD-K20168484	trt_cp
CPC011_MCF7_24H_X4_B5_DUO52HI53LO:C16	CPC011_MCF7_24H_X4	C16	BRD-K33312228	halometasone	trt_cp
CPC011_MCF7_24H_X4_B5_DUO52HI53LO:C18	CPC011_MCF7_24H_X4	C18	BRD-K44094599	tacrolimus	trt_cp
CPC005_MCF7_24H_X4_B5_DUO52HI53LO:O03	CPC005_MCF7_24H_X4	O03	BRD-K52512893	SC-19220	trt_cp
PCLB003_MCF7_24H_X1_B13:L01	PCLB003_MCF7_24H_X1	L01	BRD-K63828191	raloxifene	trt_cp
PCLB003_MCF7_24H_X2_B13:N07	PCLB003_MCF7_24H_X2	N07	BRD-K67174588	toremifene	trt_cp
CPC011_MCF7_24H_X4_B5_DUO52HI53LO:I13	CPC011_MCF7_24H_X4	I13	BRD-K01649396	indatraline	trt_cp
PCLB003_MCF7_24H_X3_B13:I13	PCLB003_MCF7_24H_X3	I13	BRD-A75409952	wortmannin	trt_cp
CPC011_MCF7_24H_X4_B5_DUO52HI53LO:M22	CPC011_MCF7_24H_X4	M22	BRD-K94649603	taxifolin	trt_cp
CPC007_MCF7_24H_X4_B5_DUO52HI53LO:A12	CPC007_MCF7_24H_X4	A12	BRD-K71746704	BRD-K71746704	trt_cp
CPC007_MCF7_24H_X2_B5_DUO52HI53LO:K19	CPC007_MCF7_24H_X2	K19	BRD-K69304050	BRD-K69304050	trt_cp
CPC013_MCF7_24H_X3_B5_DUO52HI53LO:C03	CPC013_MCF7_24H_X3	C03	BRD-K23984367	sorafenib	trt_cp
PCLB003_MCF7_24H_X3_B13:H13	PCLB003_MCF7_24H_X3	H13	BRD-K17953061	staurosporine	trt_cp
CPC011_MCF7_24H_X4_B5_DUO52HI53LO:I19	CPC011_MCF7_24H_X4	I19	BRD-K48195008	ethinylestradiol	trt_cp
PCLB003_MCF7_24H_X3_B13:E19	PCLB003_MCF7_24H_X3	E19	BRD-K21001652	BRD-K21001652	trt_cp
CPC011_MCF7_24H_X4_B5_DUO52HI53LO:C23	CPC011_MCF7_24H_X4	C23	BRD-K82109576	vincristine	trt_cp
CPC008_MCF7_24H_X5_B5_DUO52HI53LO:C20	CPC008_MCF7_24H_X5	C20	BRD-K15206810	BRD-K15206810	trt_cp
CPC011_MCF7_24H_X4_B5_DUO52HI53LO:B13	CPC011_MCF7_24H_X4	B13	BRD-A29485665	bicalutamide	trt_cp
CPC011_MCF7_24H_X4_B5_DUO52HI53LO:I15	CPC011_MCF7_24H_X4	I15	BRD-K89732114	trifluoperazine	trt_cp
CPC011_MCF7_24H_X4_B5_DUO52HI53LO:L24	CPC011_MCF7_24H_X4	L24	BRD-K39621635	artemether	trt_cp
CPC006_MCF7_24H_X4_B4_DUO52HI53LO:E22	CPC006_MCF7_24H_X4	E22	BRD-K59036917	BRD-K59036917	trt_cp
CPC006_MCF7_24H_X3_B4_DUO52HI53LO:C21	CPC006_MCF7_24H_X3	C21	BRD-K78716413	BRD-K78716413	trt_cp
CPC019_MCF7_24H_X2_F1B4_DUO52HI53LO:A05	CPC019_MCF7_24H_X2	A05	BRD-K53638321	BRD-K53638321	trt_cp
CPC006_MCF7_24H_X2_B4_DUO52HI53LO:I20	CPC006_MCF7_24H_X2	I20	BRD-K15025317	BAY-11-7082	trt_cp
PCLB003_MCF7_24H_X2_B13:A13	PCLB003_MCF7_24H_X2	A13	BRD-K03641750	FL-HDAC-025	trt_cp
CPC007_MCF7_24H_X5_B5_DUO52HI53LO:J14	CPC007_MCF7_24H_X5	J14	BRD-K03552198	BRD-K03552198	trt_cp
CPC011_MCF7_24H_X4_B5_DUO52HI53LO:H22	CPC011_MCF7_24H_X4	H22	BRD-K25224017	pirenperone	trt_cp

CPC011_MCF7_24H_X4_B5_DUO52HI53LO:K13	CPC011_MCF7_24H_X4	K13	BRD-K70778732	trazodone	trt_cp
CPD002_MCF7_24H_X1_B6_DUO52HI53LO:O24	CPD002_MCF7_24H_X1	O24	BRD-A37942872	eucatropine	trt_cp
PRISM001_MCF7_24H_X1_B7_DUO52HI53LO:G03	PRISM001_MCF7_24H_X1	G03	CMAP-AZD-1152HQPA	AZD-1152HQPA	trt_cp
CPC006_MCF7_24H_X4_B4_DUO52HI53LO:C19	CPC006_MCF7_24H_X4	C19	BRD-K77432048	BRD-K77432048	trt_cp
PRISM001_MCF7_24H_X1_B7_DUO52HI53LO:M05	PRISM001_MCF7_24H_X1	M05	BRD-K36740062	GSK-1070916	trt_cp
CPC011_MCF7_24H_X4_B5_DUO52HI53LO:B15	CPC011_MCF7_24H_X4	B15	BRD-A79903587	tegafur	trt_cp
CPC005_MCF7_24H_X4_B5_DUO52HI53LO:M24	CPC005_MCF7_24H_X4	M24	BRD-A60274948	bromocriptine	trt_cp
CPC006_MCF7_24H_X3_B4_DUO52HI53LO:I20	CPC006_MCF7_24H_X3	I20	BRD-K15025317	BAY-11-7082	trt_cp
CPC008_MCF7_24H_X4_B5_DUO52HI53LO:B14	CPC008_MCF7_24H_X4	B14	BRD-K92158425	BRD-K92158425	trt_cp
CPC008_MCF7_24H_X4_B5_DUO52HI53LO:P23	CPC008_MCF7_24H_X4	P23	BRD-K53780220	BRD-K53780220	trt_cp
CPC008_MCF7_24H_X4_B5_DUO52HI53LO:N05	CPC008_MCF7_24H_X4	N05	BRD-K45399554	CAM-9-027-3	trt_cp
CPC018_MCF7_24H_X3_B4_DUO52HI53LO:P24	CPC018_MCF7_24H_X3	P24	BRD-K15519488	CS-110266	trt_cp
CPC008_MCF7_24H_X4_B5_DUO52HI53LO:L11	CPC008_MCF7_24H_X4	L11	BRD-K44432556	VU-0418946-1	trt_cp
CPC008_MCF7_24H_X4_B5_DUO52HI53LO:G18	CPC008_MCF7_24H_X4	G18	BRD-K49183052	VU-0415113-1	trt_cp
CPC008_MCF7_24H_X4_B5_DUO52HI53LO:K17	CPC008_MCF7_24H_X4	K17	BRD-K25536815	BRD-K25536815	trt_cp
CPC008_MCF7_24H_X4_B5_DUO52HI53LO:B12	CPC008_MCF7_24H_X4	B12	BRD-K01896723	BRD-K01896723	trt_cp
CPC020_MCF7_24H_X5_B5_DUO52HI53LO:E21	CPC020_MCF7_24H_X5	E21	BRD-K94173926	I-505056	trt_cp
CPC020_MCF7_24H_X5_B5_DUO52HI53LO:N21	CPC020_MCF7_24H_X5	N21	BRD-A13188892	doxazosin	trt_cp
CPC008_MCF7_24H_X4_B5_DUO52HI53LO:B24	CPC008_MCF7_24H_X4	B24	BRD-K36153907	KU-C103885	trt_cp
CPC020_MCF7_24H_X5_B5_DUO52HI53LO:A15	CPC020_MCF7_24H_X5	A15	BRD-K08339178	BRD-K08339178	trt_cp
CPC008_MCF7_24H_X4_B5_DUO52HI53LO:O24	CPC008_MCF7_24H_X4	O24	BRD-K95337198	BRD-K95337198	trt_cp
CPC008_MCF7_24H_X4_B5_DUO52HI53LO:P19	CPC008_MCF7_24H_X4	P19	BRD-K77681376	BRD-K77681376	trt_cp
CPC020_MCF7_24H_X5_B5_DUO52HI53LO:O15	CPC020_MCF7_24H_X5	O15	BRD-K52522949	NCH-51	trt_cp
CPC020_MCF7_24H_X5_B5_DUO52HI53LO:A12	CPC020_MCF7_24H_X5	A12	BRD-A09722536	cyclophosphamide	trt_cp
CPC008_MCF7_24H_X4_B5_DUO52HI53LO:L17	CPC008_MCF7_24H_X4	L17	BRD-K05563014	CAM-9-021	trt_cp
CPC016_MCF7_24H_X2_B4_DUO52HI53LO:B20	CPC016_MCF7_24H_X2	B20	BRD-A82656074	naltrindole	trt_cp
CPC008_MCF7_24H_X4_B5_DUO52HI53LO:G20	CPC008_MCF7_24H_X4	G20	BRD-K39318886	BRD-K39318886	trt_cp
CPC008_MCF7_24H_X4_B5_DUO52HI53LO:P21	CPC008_MCF7_24H_X4	P21	BRD-K74777906	BRD-K74777906	trt_cp

MUC.CP002_MCF7_24H_X2_B7_DUO52HI53LO:C19	MUC.CP002_MCF7_24H_X2	C19	BRD-K28936863	ketotifen	trt_cp
MUC.CP001_MCF7_24H_X2_B7_DUO52HI53LO:A04	MUC.CP001_MCF7_24H_X2	A04	BRD-A75409952	wortmannin	trt_cp
MUC.CP002_MCF7_24H_X2_B7_DUO52HI53LO:I19	MUC.CP002_MCF7_24H_X2	I19	BRD-A22305049	benfluorex	trt_cp
MUC.CP002_MCF7_24H_X2_B7_DUO52HI53LO:K07	MUC.CP002_MCF7_24H_X2	K07	BRD-K98548675	parthenolide	trt_cp
MUC.CP001_MCF7_24H_X2_B7_DUO52HI53LO:I07	MUC.CP001_MCF7_24H_X2	I07	BRD-K45435259	SCH-23390	trt_cp
MUC.CP002_MCF7_24H_X2_B7_DUO52HI53LO:G17	MUC.CP002_MCF7_24H_X2	G17	BRD-K77987382	mebendazole	trt_cp
CPC019_MCF7_24H_X3_F2B4_DUO52HI53LO:L22	CPC019_MCF7_24H_X3	L22	BRD-K45232279	BRD-K45232279	trt_cp
MUC.CP001_MCF7_24H_X2_B7_DUO52HI53LO:O09	MUC.CP001_MCF7_24H_X2	O09	BRD-A21329147	hemicholinium-3	trt_cp
CPC020_MCF7_24H_X4_F2B4_DUO52HI53LO:I21	CPC020_MCF7_24H_X4	I21	BRD-K32112425	BRD-K32112425	trt_cp
MUC.CP002_MCF7_24H_X2_B7_DUO52HI53LO:K19	MUC.CP002_MCF7_24H_X2	K19	BRD-K94325918	kinetin-riboside	trt_cp
MUC.CP002_MCF7_24H_X2_B7_DUO52HI53LO:K09	MUC.CP002_MCF7_24H_X2	K09	BRD-K35424586	SA-247714	trt_cp
CPC006_MCF7_24H_X4_B4_DUO52HI53LO:E16	CPC006_MCF7_24H_X4	E16	BRD-A02006392	nitrendipine	trt_cp
CPC014_MCF7_24H_X1_B5_DUO52HI53LO:O19	CPC014_MCF7_24H_X1	O19	BRD-K32927008	BRD-K32927008	trt_cp
CPC019_MCF7_24H_X3_F2B4_DUO52HI53LO:J22	CPC019_MCF7_24H_X3	J22	BRD-K63979920	BRD-K63979920	trt_cp
CPC005_MCF7_24H_X1_B4_DUO52HI53LO:A05	CPC005_MCF7_24H_X1	A05	BRD-A45889380	mepacrine	trt_cp
CPD002_MCF7_24H_X3_B6_DUO52HI53LO:P12	CPD002_MCF7_24H_X3	P12	BRD-K32021043	sulfachlorpyridazine	trt_cp
ASG001_MCF7_24H_X1_B7_DUO52HI53LO:A04	ASG001_MCF7_24H_X1	A04	BRD-A19037878	trichostatin-a	trt_cp
MUC.CP001_MCF7_24H_X2_B7_DUO52HI53LO:M11	MUC.CP001_MCF7_24H_X2	M11	BRD-K17025677	BRD-K17025677	trt_cp
CPC005_MCF7_24H_X4_B5_DUO52HI53LO:K09	CPC005_MCF7_24H_X4	K09	BRD-A79226577	tropisetron	trt_cp
CPC014_MCF7_24H_X1_B5_DUO52HI53LO:I14	CPC014_MCF7_24H_X1	I14	BRD-A61666925	EMF-sumo1-6	trt_cp
